# Development and external validation of multivariable risk models to predict incident and resolved neuropathic pain: a DOLORisk Dundee study

**DOI:** 10.1007/s00415-022-11478-0

**Published:** 2022-11-10

**Authors:** Harry L. Hébert, Abirami Veluchamy, Georgios Baskozos, Francesca Fardo, Dimitri Van Ryckeghem, Ewan R. Pearson, Lesley A. Colvin, Geert Crombez, David L. H. Bennett, Weihua Meng, Colin N. A. Palmer, Blair H. Smith

**Affiliations:** 1 Chronic Pain Research Group, Division of Population Health and Genomics, Mackenzie Building, Ninewells Hospital and Medical School, University of Dundee, Kirsty Semple Way, Dundee, DD2 4BF UK; 2 Pat Macpherson Centre for Pharmacogenetics and Pharmacogenomics, Division of Population Health and Genomics, Ninewells Hospital and Medical School, University of Dundee, Dundee, UK; 3 Neural Injury Group, Nuffield Department of Clinical Neuroscience, John Radcliffe Hospital, University of Oxford, Oxford, UK; 4 Danish Pain Research Center, Department of Clinical Medicine, Aarhus University, Aarhus, Denmark; 5 Department of Experimental-Clinical and Health Psychology, Faculty of Psychology and Educational Sciences, Ghent University, Ghent, Belgium; 6 Section Experimental Health Psychology, Clinical Psychological Science, Departments, Faculty of Psychology and Neuroscience, Maastricht University, Maastricht, Netherlands

**Keywords:** Neuropathic pain, Prediction model, Risk factors, Epidemiology, Scotland, DOLORisk, Validation

## Abstract

Neuropathic pain is difficult to treat, and an understanding of the risk factors for its onset and resolution is warranted. This study aimed to develop and externally validate two clinical risk models to predict onset and resolution of chronic neuropathic pain. Participants of Generation Scotland: Scottish Family Health Study (GS; general Scottish population; *n = *20,221) and Genetic of Diabetes Audit and Research in Tayside Scotland (GoDARTS; *n = *5236) were sent a questionnaire on neuropathic pain and followed- -up 18 months later. Chronic neuropathic pain was defined using DN4 scores (≥ 3/7) and pain for 3 months or more. The models were developed in GS using logistic regression with backward elimination based on the Akaike information criterion. External validation was conducted in GoDARTS and assessed model discrimination (ROC and Precision-Recall curves), calibration and clinical utility (decision curve analysis [DCA]). Analysis revealed incidences of neuropathic pain onset (6.0% in GS [236/3903] and 10.7% in GoDARTS [61/571]) and resolution (42.6% in GS [230/540] and 23.7% in GoDARTS [56/236]). Psychosocial and lifestyle factors were included in both onset and resolved prediction models. In GoDARTS, these models showed adequate discrimination (ROC = 0.636 and 0.699), but there was evidence of miscalibration (Intercept = − 0.511 and − 0.424; slope = 0.623 and 0.999). The DCA indicated that the models would provide clinical benefit over a range of possible risk thresholds. To our knowledge, these are the first externally validated risk models for neuropathic pain. The findings are of interest to patients and clinicians in the community, who may take preventative or remedial measures.

**Supplementary Information:**

The online version contains supplementary material available at 10.1007/s00415-022-11478-0.

## Introduction

Neuropathic pain is a common and unpleasant form of pain arising from a lesion or disease affecting the somatosensory nervous system [1]. It is estimated that up to 20% of individuals with chronic pain (usually taken as pain lasting more than 3 months [2]) suffer from pain with neuropathic characteristics [3]. The prevalence in the general population is 7–10% [4]. Neuropathic pain is often described with terms such as “burning”, “electric shock” and “pins and needles” and about 17% rate their quality of life as “worse than death” [3]. The disorder is associated with reduced employability, increased social isolation and high dependence on primary care services [5]. However, not everyone with a relevant lesion or disease develops neuropathic pain. For example, it is estimated that only 10–26% of those with type 2 diabetes develop neuropathic pain (painful diabetic neuropathy) [4, 6].

Neuropathic pain can be difficult to manage. Many common analgesics used to treat nociceptive pain, including opioids, are of limited benefit in neuropathic pain. Furthermore, first-line medications for neuropathic pain including gabapentinoids (e.g. gabapentin/pregabalin), tricyclic antidepressants (e.g. amitriptyline) and serotonin-norepinephrine reuptake inhibitors (e.g. duloxetine), provide greater than 50% pain relief in less than 50% of those treated [7].

Epidemiological approaches have been used to explain the variability in the onset and prognosis of neuropathic pain onset. Neuropathic pain is considered a complex disorder with both genetics and environmental factors playing a role in its development [8, 9]. A recent twins study reported that environmental factors account for 63% of the variability in neuropathic pain onset [10]. Moreover, a number of psychological comorbidities have been associated with neuropathic pain, including sleep disturbance, anxiety and depression [11], as well as demographic factors such as older age [12] and female gender [13]. However, the majority of epidemiological studies in neuropathic pain have been cross-sectional in design and, therefore, unable to establish the temporal relationship between potential explanatory factors and outcome [14].

Clinical prediction models use individual patient data to estimate the probability that a certain health outcome will occur and can help inform decision making processes [15]. They can be used for a variety of purposes, including predicting the onset of disease, response to treatment or prognosis. In the context of neuropathic pain, prediction models could be useful for identifying which patients are likely to develop the disorder, thus enabling clinicians to take more targeted preventative measures. Alternatively, clinical models could be used to mitigate against persistence in those already with neuropathic pain, by identifying factors that predict its resolution. To the best of our knowledge, there are no such externally validated clinical prediction models for neuropathic pain.

The aim of this study was to develop and externally validate the following two clinical prediction models for neuropathic pain: one to predict risk of incident neuropathic pain and one to predict resolution of neuropathic pain.

## Methods

We followed the Transparent Reporting of a Multivariable Prediction Model for Individual Prognosis or Diagnosis (TRIPOD) statement for the reporting of this study (Supplementary Table S1) [16].

### Study design

This prospective study is part of DOLORisk (http://dolorisk.eu/), a multicentre collaboration which aims to understand the risk factors and determinants of neuropathic pain [17]. This branch of the study (DOLORisk Dundee) has been described in detail elsewhere [18].

In brief, data were used from two Scottish population cohorts. Generation Scotland: Scottish Family Health Study (GS; [19, 20]) was used as a development cohort, whereas the Genetics of Diabetes Audit and Research in Tayside Scotland (GoDARTS; [21]) was used as a validation cohort. Validation of a prediction model in independent cohorts (i.e. external validation) is necessary to maximise the generalizability to different risk populations and to help mitigate against overfitting (producing a model that too closely resembles the data that were used to develop it and fails to explain additional data). Due to possible overfitting, validation of a prediction model within the same cohort (i.e. internal validation) has typically limited generalizability because a model tends to perform well in the cohort that was used to develop it.

GS is a family-based cohort, consisting of 24,084 volunteers (aged 18–98 years at GS baseline) recruited from primary care centres across Scotland between 2006 and 2011. GoDARTS is a population cohort consisting of 10,149 participants with diabetes (predominantly type 2) and 8,157 diabetes-free controls recruited from the Tayside region of Scotland between 1998 and 2015. Both cohorts are predominantly white/Caucasian (> 95%), whilst GoDARTS is older (median: 71 years vs 60 years) and has a higher proportion of males (61% vs 39%) compared to GS. As part of these individual studies, demographic, health and lifestyle data are available from questionnaires completed at recruitment. Furthermore, in GS biochemical data are available from blood and urine samples taken around the same time as the questionnaire. In GoDARTS, biochemical and clinical data are available through electronic record linkage to routinely collected longitudinal National Health Service (NHS) data.

### Recruitment

As both development and validation cohorts lacked data on neuropathic pain, participants of both cohorts who were still alive, residing in Scotland and had provided consent to be re-contacted about further studies, were sent and invited to return a paper questionnaire by post between May and December 2016 (GS = 20,221; GoDARTS = 5236). The baseline questionnaire included validated screening tools assessing health-related quality of life (HRQoL; EuroQoL-five dimensions five levels [EQ5D-5L] [22]) depression [23], anxiety [23] and sleep disturbance [24] (all PROMIS SF-4a), personality dimensions (Ten Item Personality Inventory [TIPI] [25]) and pain-related worrying (pain catastrophising scale [PCS]) [26]. In addition to these tools, other domains including adverse childhood experiences, smoking and alcohol history were assessed using ad hoc questions developed for DOLORisk. Participants were also asked to complete two pain screening questions on current pain (‘Are you currently troubled by pain or discomfort, either all the time or on and off?’) and current pain medication (‘Are you currently taking medications specifically to treat pain or discomfort?’), with the former having been used in the original GS study recruitment [27] and the latter used in a GS and 23andMe study collaboration [28]. Those responding ‘yes’ to either question were then asked to complete further pain related items including duration and the seven-item self-report version of the neuropathic pain screening tool *Douleur Neuropathique en Quatre Questions* (DN4) [29].

After 18 months, those who returned completed questionnaires, provided further consent to be re-contacted, and who had not died in the interim (GS = 6657; GoDARTS = 1460), were invited to complete a follow-up questionnaire online or sent a paper questionnaire by post, depending on whether an email address had been provided. The follow-up questionnaire was a reduced version of the baseline questionnaire and did not contain items relating to adverse childhood experiences, personality traits, smoking or alcohol. Further details on the baseline and follow-up questionnaires can be found in the study profile papers [17, 18].

### Outcome

Two models were developed for this study. The aim of the first model was to predict new cases of chronic neuropathic pain at follow-up (incident neuropathic pain). The second model aimed to predict resolved chronic neuropathic pain at follow-up. To be considered as having chronic neuropathic pain, a participant needed to satisfy the following criteria:

1. Answered in the affirmative to either ‘Are you currently troubled by pain or discomfort, either all the time or on and off?’ or ‘Are you currently taking medications specifically to treat pain or discomfort?’
2. Have a self-complete *Douleur Neuropathique en 4 Questions* (DN4) score of 3 or more out of 7 [29].
3. Reported a pain duration of 3 months or more.

Participants were considered to have no neuropathic pain if they did not meet the above criteria. Participants were excluded from analysis if they could not be classified for 1–2 as a result of missing data or if they satisfied 2, but not 1 and/or 3.

### Predictors

Potential predictors were chosen based on past literature reviews of risk factors for neuropathic pain and availability in both GS and GoDARTS (Table 1) [30–32]. Psychological and lifestyle factors (apart from physical activity) were all derived from the baseline questionnaire. Demographic, clinical and biomarker data were all derived from pre-existing cohort data from GoDARTS and GS. The demographic data included a variable for the level of social deprivation, linked to each participants’ home postal code, as measured by the Scottish Index of Multiple Deprivation (SIMD) [33]. The SIMD assesses social deprivation in 6,976 geographical areas in Scotland using seven domains: income, employment, health, crime, education, housing and access to services (e.g. GP practice, post office and schools). Each geographical area is ranked according to the composite of these domains (1 being most deprived and 6,976 being least deprived). For the purposes of analysis, the rankings were divided into quintiles of equal size with rank 1 being most deprived (top 20%) and rank 5 being least deprived (bottom 20%). Where clinical and biomarker predictors were available longitudinally through electronic record linkage in GoDARTS (as well as social deprivation and physical activity), we used the most recent value preceding the start of the baseline survey (May 2016). Data derived from GS were obtained at study recruitment from 2006 to 2011. Continuous variables were dichotomised into two categories to facilitate the interpretation of the two models. Dichotomisation was based either on clinically meaningful boundaries for clinical and biomarker predictors (e.g. BMI ≥ 30 kg/m^2^ for obesity) or consensus-based clinically relevant cut-offs (e.g. PCS ≥ 30 for pain-related worrying [26]). The following potential predictors were initially entered into the analysis for both models (Table 1):

- Age (< 65 years, ≥ 65 years)
- Sex (male/female).
- Social deprivation (Scottish Index of Multiple Deprivation [SIMD] Quintiles)
- Depression, anxiety and sleep disturbance (PROMIS Short Form-4a [SF-4a]; *T*-Score: < 50, ≥ 50 [23, 24])
- Extraversion, agreeableness, conscientiousness, emotional stability and being open to new experiences (Ten Item Personality Inventory [TIPI]; < 5, ≥ 5 [25])
- Pain-related worrying (PCS < 30, ≥ 30 [26])
- Adverse childhood experiences (ACE; ‘Before the age of 18, have you ever experienced severe traumatic events?’)
- Hospital stay before 18 years of age (‘Before the age of 18, have you ever stayed in hospital for a long period because of a life-threatening disease or situation?’)
- Ever smoked (‘Have you ever been a regular smoker of tobacco?’)
- Currently drink alcohol (‘On average how often do you currently drink alcohol?’)
- Physical activity (high/low)
- HRQoL (EuroQoL 5 Dimensions, 5 Levels [EQ5D-5L]; utility index: < 0.800, ≥ 0.800 [22])
- Body mass index (BMI) (< 30 kg/m^2^, ≥ 30 kg/m^2^ [obesity])
- Resting heart rate (< 100 beats per minute [BPM], ≥ 100 BPM [tachycardia])
- Blood pressure (< 140/90 mmHg, ≥ 140/90 mmHg [hypertension])
- Coronary artery disease (CAD)/Heart disease (GoDARTS–International Classification of Disease, 10.^th^ revision [ICD-10] codes I20-I25, GS–self-reported)
- Stroke (GoDARTS – ICD-10 codes I60-61 and I63-64, GS–self-reported)
- Fasting glucose (< 7 mmol/L, ≥ 7 mmol/L [hyperglycemia])
- Serum creatinine (< 100 µmol/L, ≥ 100 µmol/L)
- Total cholesterol (< 5 mmol/L, ≥ 5 mmol/L)
- High–density lipoprotein [HDL] (< 1 mmol/L, ≥ 1 mmol/L)

**Table 1:** Candidate predictors included in the model development process

Group	Characteristic	Measure	Source	Cut-off	References
Demographic	Age	Years	Cohorts	< 65, ≥ 65	n/a
	Gender	Male/Female	Cohorts	n/a	n/a
	Social deprivation	SIMD Quintiles	Cohorts	n/a	n/a
Psychological	Depression	PROMIS SF4a	Questionnaire	*T*-Score: < 50, ≥ 50	Pilkonis et al. [39]
	Anxiety	PROMIS SF4a	Questionnaire	*T*-Score: < 50, ≥ 50	Pilkonis et al. [39]
	Sleep disturbance	PROMIS SF4a	Questionnaire	*T*-Score: < 50, ≥ 50	Yu et al. [71]
	Extraversion	TIPI	Questionnaire	Score: < 5, ≥ 5	Gosling et al. [20]
	Agreeableness	TIPI	Questionnaire	Score: < 5, ≥ 5	Gosling et al. [20]
	Conscientiousness	TIPI	Questionnaire	Score: < 5, ≥ 5	Gosling et al. [20]
	Emotional stability	TIPI	Questionnaire	Score: < 5, ≥ 5	Gosling et al. [20]
	Open to new experiences	TIPI	Questionnaire	Score: < 5, ≥ 5	Gosling et al. [20]
	Pain catastrophising	PCS	Questionnaire	Score: < 30, ≥ 30	Sullivan et al. [56]
	Traumatic events before 18 years of age	Yes/No	Questionnaire	n/a	n/a
	Hospital stay before 18 years of age	Yes/No	Questionnaire	n/a	n/a
Lifestyle	Ever smoked	Yes/No	Questionnaire	n/a	n/a
	Currently drink alcohol	Yes/No	Questionnaire	n/a	n/a
	Physical activity	High/Low	Cohorts	n/a	n/a
	Health-related quality of life	EQ5D-5L	Questionnaire	Index: < 0.800, ≥ 0.800	EuroQol-Group [12]
Clinical	BMI	Kg/m^2^	Cohorts	< 30, ≥ 30	n/a
	Resting heart rate	BPM	Cohorts	< 100, ≥ 100	n/a
	Blood pressure	mmHg	Cohorts	< 140/90, ≥ 140/90	n/a
	Coronary artery disease/heart disease	ICD-10 codes I20-25/self-reported^a^	Cohorts	n/a	n/a
	Stroke	ICD-10 codes I60-61, I63-64/self-reported^a^	Cohorts	n/a	n/a
Biomarkers	Glucose	mmol/L	Cohorts	< 7, ≥ 7	n/a
	Creatinine	µmol/L	Cohorts	< 100, ≥ 100	n/a
	Total cholesterol	mmol/L	Cohorts	< 5, ≥ 5	n/a
	High density lipoprotein	mmol/L	Cohorts	< 1, ≥ 1	n/a

*BMI* body mass index; *BPM* beats per minute; *EQ-5D-5L* EuroQoL-five dimensions-five levels; *ICD* International Classification of Diseases; *PCS* Pain Catastrophising Scale; *PROMIS* patient-reported outcomes measurement information system; *SF4a* short form four answers; *SIMD* Scottish Index of Multiple Deprivation; *TIPI* ten item personality inventory

^a^GoDARTS/GS:SFHS

Uninformative predictors with low variance can cause problems with model convergence and reduce statistical power in model development. Therefore, all potential predictors with zero or near-zero variance, defined as the ratio of the most common group to the second most common group of more than 19, were removed from analysis [34].

### Sample size

An important factor in model development is the events per variable (EPV) ratio. For a binary outcome, the number of ‘events’ is the smaller of the number of participants who experienced the outcome (e.g. incident or resolved neuropathic pain) and the number of participants who did not experience the outcome. The number of ‘variables’ is the number of degrees of freedom required to represent all of the predictors in the model.

To avoid overfitting a model, different minimum EPV ratios have been proposed as a general rule of thumb, but they commonly fall in the range of 5–15 [35–37]. After removing predictors with near zero variance, the EPV ratio was 8.2 for incident neuropathic pain and 7.1 for resolved neuropathic pain.

### Missing data

Missing data in predictor variables are common in epidemiology studies. Missing data can cause problems with statistical analysis and lead to unreliable results. A simple solution is to perform a complete case analysis by only retaining participants who are non-missing for all predictors being analysed. However, this approach reduces the statistical power of the study by removing useful information from participants with incomplete data. It also assumes that there are no differences in the observed predictors between participants with and without missing data (i.e. that the data are missing completely at random), which can introduce bias if violated. An alternative approach is to replace the missing values using multiple imputation (MI), thereby retaining participants with missing data and conserving statistical power. MI also considers the uncertainty introduced by imputations by performing the analysis *m* times creating *m* datasets.

MI was performed in both GS (for model development and internal validation) and GoDARTS (for external validation) using multivariate imputation by chained equations. Social deprivation was imputed using ordinal regression and all other predictors were imputed using binary regression. The number of imputed datasets created was equal to the percentage of participants with at least one missing observation [38]. All independent variables were used as predictors in the imputation models as well as being imputed themselves. The outcome variable was used as a predictor in the imputation but was not imputed itself.

### Statistical analysis

Baseline descriptive statistics for both GS and GoDARTS were reported with continuous variables presented as the median and interquartile range and categorical variables presented as percentages.

The GS cohort was split randomly into two separate datasets, one for model development (80%) and one for internal validation (20%). After removing predictors with near-zero variance and MI, all remaining variables were entered into the analysis. Initially, univariate analysis was conducted in each imputed dataset in the development cohort using logistic regression, and the results were pooled using Rubin’s rules. Then multivariate analysis was conducted to identify independent predictors using logistic regression with backward elimination in each imputed dataset. Any predictor appearing in at least 50% of the models was then carried forward to the next stage. The final model was obtained by re-running the logistic regression with the selected predictors in all the imputed datasets and pooling the results, as before, to obtain the model parameters [39].

A key assumption of logistic regression is that all predictors should be independent (i.e. low collinearity or correlation between predictors). To ensure that the analysis did not violate the collinearity assumption, the variance inflation factor (VIF) was calculated for each predictor in the final model, with higher values indicating increased inflation of the standard error of the predictor. A VIF of less than 3 is considered an acceptable level of collinearity [40].

To assess the goodness of fit, a likelihood ratio (LR) test was conducted to compare the models to an intercept-only (null) model. This assesses whether the developed model can explain the data better than the nested null model. The null hypothesis is that there is no difference in the likelihoods (i.e. the LR is close to 1) and the developed model is no better at explaining the data than the null model. Model performance was also analysed by calculating the Nagelkerke *R*^2^, which measures the additional proportion of the variance in the outcome that is explained by the developed model compared to the null model. Nagelkerke *R*^2^ ranges from 0 to 1, with higher values indicating a greater proportion of the variance in the outcome [41].

Internal (GS validation set) and external validation (GoDARTS) consisted of analysing model discrimination and calibration by calculating risk scores for each participant according to the model algorithm. Additionally, the clinical utility of each model was analysed during external validation [42]. Discrimination refers to the ability of a model to correctly separate individuals who experience an event (in this case incident or resolved neuropathic pain) from those that do not. A model with good discrimination should assign higher probability scores to individuals with the outcome and lower probability scores to those without the outcome, with minimal overlap between the two groups. Model discrimination was assessed by plotting the receiver operating characteristic (ROC) curve and calculating the area under the cure (AUROC) in each of the imputed datasets. The ROC curve depicts the true positive rate versus the false positive rate over a range of probability thresholds. The AUROC, also known as the C-statistic, ranges from 0.5 for a model that discriminates no better than chance, to 1 for a model that demonstrates perfect discrimination, with 0.5–0.6 defined as “poor”, 0.6–0.7 defined as “acceptable”, 0.7–0.8 defined as good, 0.8–0.9 as “very good” and > 0.9 defined as “excellent” [43].

When there is an imbalance of positive and negative observations in the outcome being predicted, the AUROC can be misleading with respect to model discrimination [44]. The reason for this is that the greatly increased number of negative outcomes results in very little fluctuation in the false positive rate and, therefore, overall AUROC. This is the case, for example, in diseases with low prevalence/incidence, where those without the disease (negative class) far outweigh those with the disease (the minority class). An alternative measure of model discrimination is the precision-recall curve (PRC), which plots the positive predictive value (precision) against the true positive rate (recall) over a range of probability thresholds. Unlike the ROC curve, the PRC does not consider the number of true negative observations and is, therefore, more sensitive to class imbalance. The PRC was plotted for each imputed dataset and the corresponding area under the PRC metric (AUPRC) was calculated. The performance of the AUPRC is assessed relative to the incidence of the positive outcome, with a value greater than this indicating that the model is discriminating better than chance and higher values indicating better discrimination (up to 1) [44].

Calibration relates to how well a model’s predicted risk for a particular outcome compares to the observed risk in the dataset [42]. If 100 people are predicted by a model to have a 20% chance of experiencing an outcome (i.e. predicted risk), then 20 of those people would be expected to experience the outcome (i.e. observed risk). Model calibration was assessed graphically by plotting a line of regression of the response variable on the logit of predicted probabilities from the models in each of the imputed datasets. A model with perfect calibration is indicated by a regression curve on the 45° line with a corresponding slope of 1 and intercept of 0. A slope below 1 and intercept below 0 indicates overfitting of the model, whereas the opposite indicates underfitting of the model. Both models were updated using the logistic recalibration method on the external validation set [42]. In this approach, the regression coefficients (including intercept) in the model algorithms were multiplied by a factor equal to the sum of the corresponding calibration intercept and slope.

Clinical utility addresses how well a model performs when it is used in the context of patient care and, therefore, adds information to the statistical accuracy which is offered by discrimination and calibration analysis. For example, a treatment may be available for a particular outcome with associated benefits in treating patients with the outcome (true positives) and harms in unnecessarily treating patients without the outcome (false positives). Often the decision on whether to administer the treatment to someone is based on the probability of them experiencing the outcome. This comparison can be described with respect to a particular prediction model in terms of the “net benefit”, which measures the difference in proportions between true positives (TP) and false positives (FP) at a particular probability threshold (Pt). Net benefit can be calculated using the following equation:

$${\text{Net}}\,{\text{ Benefit}}\, = \frac{TP}{n} - \frac{FP}{n}\left( {\frac{Pt}{{1 - Pt}}} \right),$$

Where *n* is the total number of subjects. A key concept is that the probability threshold (representing a hypothetical cut-off for deciding whether to initiate treatment) is used to inform the relative importance of true positives and false positives, with the odds ratio being used to weight the harm of false positives. Low probability thresholds (Pt < 50%) indicate that the benefits of treating positive cases outweigh the harms of treating negative cases and vice versa. Clinical utility was assessed by way of a decision curve analysis (DCA) of net benefit over a range of probability thresholds [45]. This was used to compare interventions guided by the developed models to a “treat all” approach where everyone is given the intervention and a “treat none” approach where nobody is given the intervention. The net benefit of a “treat none” approach is by definition zero across all threshold probabilities and the “treat all” approach crosses the y axis (zero probability) and the x-axis (zero net benefit) at the incidence of outcome. A model demonstrating perfect prediction will have a net benefit at the incidence of outcome across all probability thresholds. The best model is the one with the highest net benefit over the full range of threshold probabilities. A prediction model usually has the highest net benefit around the probability threshold equal to the incidence of outcome and therefore this was used to summarise the DCA. In this study, “treat all” refers to the initiation of treatment to prevent neuropathic pain onset or the continuation of treatment in existing neuropathic pain to prevent persistence.

All statistical analyses were conducted in R v.3.5.3 [46]. MI was conducted using the MICE package [47], ROC curves and calibration plots were constructed using the modEvA package [48], PRCs were constructed using the PRROC package [49, 50] and DCAs were constructed using the rmda package [51]. To present the ROC curve, PRC, calibration plot and DCA, a dataset was selected at random by R from those produced from MI. Overall validation measures for model discrimination, calibration and clinical utility were presented as the median and interquartile range (IQR).

### Patient and public involvement

No patients were directly involved in the design or implementation of this study. Furthermore, no patients were involved in the interpretation of results or the drafting of this paper. However, DOLORisk is linked to several patient support organisations, who contributed to its overall design and implementation, and the results of this study will be disseminated to the wider patient community.

## Results

### Participant flow

A detailed flow of participants in the two cohorts has previously been published [18]. The baseline response rate was 35.8% (7240/20,221) in GS and 36.6% in GoDARTS (1915/5236). Of those who provided consent to be contacted again and had not subsequently withdrawn or died, the response rate to the follow-up survey was 79.5% (5292/6657) in GS and 71.6% in GoDARTS (1046/1460). The overall response rate (participants who responded to both surveys) was 26.2% (5292/20,221) in GS and 20.0% (1046/5236) in GoDARTS.

### Baseline characteristics

- Incident neuropathic pain

Of the participants who completed both questionnaires, in GS 4197 had no neuropathic pain at baseline and in GoDARTS 640 had no neuropathic pain at baseline. These participants were considered for inclusion in the development and validation of the risk model for incident neuropathic pain. The baseline characteristics of the two cohorts considered for this model, including percentage of missing data for each variable, are provided in Table 2. Compared to those from GoDARTS, included participants from GS were younger (median: 60 years vs 70 years), had a lower proportion of males (40% vs 63%) and had a higher proportion of participants in the “least deprived” category of the SIMD (40% vs 23%). In terms of lifestyle factors, GS had a lower proportion of included participants who had ever smoked (35% vs 54%) and a lower proportion of included participants who were rated in the high category of physical activity (48% vs 90%), whilst those included in GoDARTS had a lower proportion of current drinkers (78% vs 91%). The proportion of included people with CAD or heart disease was lower in GS (3% vs 16%) and unsurprisingly, the glucose levels were higher in those from GoDARTS (8.0 vs 4.7 mmol/L). Psychological measures had similar scores in both cohorts.

(b) Resolved neuropathic pain

**Table 2:** Baseline characteristics of participants who were considered for inclusion in the development (GS:SFHS) and validation (GoDARTS) of the risk model for incident neuropathic pain

Characteristic	GS:SFHS (*n = *4,197)		GoDARTS (*n = *640)	
	Observations^a^	Missing data	Observations^a^	missing data
Incidence of neuropathic pain at follow-up, *n* (%)	236 (6.0)	7	61 (10.7)	11
Age (years)	60 (50–66)	0	70 (63–76)	< 1
Gender (% male)	40	0	63	< 1
Social deprivation (SIMD; %)				
1 (most deprived)	6	12	10	36
2	10		17	
3	16		20	
4	28		30	
5 (least deprived)	40		23	
Depression (PROMIS SF4a *T*-score)	41.0 (41.0–51.8)	4	41.0 (41.0–51.8)	7
Anxiety (PROMIS SF4a *T*-score)	40.3 (40.3–51.2)	4	40.3 (40.3–51.2)	6
Sleep disturbance (PROMIS SF4a *T*-score)	46.2 (41.1–52.4)	7	48.4 (41.1–54.3)	13
Extraversion (TIPI score)	4 (3–5.5)	3	4 (3–5)	7
Agreeableness (TIPI score)	5.5 (4.5–6.5)	3	5 (4.5–6.5)	5
Conscientiousness (TIPI score)	6 (5.5–7)	3	6 (5–6.625)	6
Emotional stability (TIPI score)	5.5 (4–6.5)	3	5.5 (4–6.5)	5
Open to new experiences (TIPI score)	5 (4–6)	3	5 (4–5.5)	5
Pain catastrophising (PCS score)	3 (0–8)	3	3 (0–8)	6
Traumatic events before 18 years of age (%)	30	2	24	4
Hospital stay before 18 years of age (%)	6	5	10	8
Ever smoked (%)	35	< 1	54	1
Currently drink alcohol (%)	91	< 1	78	1
Physical activity (%; high)	48	42	90	20
Health-related quality of life (EQ-5D-5L Index)	0.837 (0.768–1.000)	4	0.837 (0.710–1.000)	6
BMI (kg/m^2^)	25.39 (22.82–28.33)	8	29.675 (26.6–33.4)	0
Resting heart rate (BPM)	68 (61–75)	5	71 (62.5–80.5)	36
Hypertension (%)	34	8	30	0
Coronary artery disease/heart disease (%)	3	8	16	0
Stroke (%)	< 1	8	3	0
Glucose (mmol/L)	4.7 (4.4–5.0)	10	8.0 (6.6–11.225)	41
Creatinine (µmol/L)	71 (63–82)	9	78 (64–92)	< 1
Total cholesterol (mmol/L)	5.2 (4.5–5.9)	9	4.00 (3.30–4.32)	< 1
High Density Lipoprotein (mmol/L)	1.5 (1.2–1.8)	9	1.00 (0.94–1.36)	< 1

*BMI* body mass index; *BPM* beats per minute; *EQ-5D-5L* EuroQoL-five dimensions-five levels; *GoDARTS* Genetics of Diabetes Audit and Research in Tayside Scotland; *GS:SFHS* Generation Scotland: Scottish Family Health Study; *PCS* Pain Catastrophising Scale; *PROMIS* patient-reported outcomes measurement information system; *SF4a* short form four answers; *SIMD* Scottish Index of Multiple Deprivation; *TIPI* ten item personality inventory

^a^Observations reported as percentages or median and interquartile range, excluding missing data

Of the participants who completed both questionnaires, in GS 632 had neuropathic pain at baseline and in GoDARTS 268 had neuropathic pain at baseline. These participants were considered for inclusion in the development and validation of the risk model for neuropathic pain resolution. The baseline characteristics of the two cohorts considered for this model are provided in Table 3. Similar to the model for incident neuropathic pain, participants in the model for resolved neuropathic pain were younger (median: 60 years vs 67 years), had a lower proportion of males (34% vs 55%) and had a higher proportion of participants in the least deprived category of the SIMD (29% vs 16%) in GS compared to GoDARTS. Similarly, GS included a lower proportion of participants who had ever smoked (49% vs 60%), a higher proportion of current drinkers (84% vs 62%) and a lower proportion of participants in the high category of physical activity (54% vs 79%). CAD/heart disease was less prevalent among those included from GS (5% vs 29%) and the glucose levels were lower compared to those from GoDARTS (median: 4.7 vs 8.0 mmol/L). In addition, there was a notable difference in the scores for the PROMIS items relating to depression (51.8 vs 55.7), anxiety (51.2 vs 53.7) and sleep disturbance (54.3 vs 56.1), as well as the PCS score (9 vs 15.5), which were lower in GS compared to GoDARTS.

**Table 3:** Baseline characteristics of the participants who were included in the development (GS:SFHS) and validation (GoDARTS) of the risk model for resolved neuropathic pain

Characteristic	GS:SFHS (*n = *632)		GoDARTS (*n = *268)	
	Observations^a^	Missing data	Observations^a^	Missing data
Remission of neuropathic pain at follow-up, *n* (%)	230 (42.6)	15	56 (23.7)	14
Age (years)	60 (52–66)	0	67 (61–75)	< 1
Gender (% male)	34	0	55	< 1
Social deprivation (SIMD; %)				
1 (Most deprived)	16	11	21	25
2	17		17	
3	15		16	
4	24		29	
5 (Least deprived)	29		16	
Depression (PROMIS SF4a *T*-score)	51.8 (41.0–58.9)	5	55.7 (49.0–62.2)	7
Anxiety (PROMIS SF4a *T*-score)	51.2 (40.3–59.5)	6	53.7 (48.0–61.4)	10
Sleep disturbance (PROMIS SF4a *T*-score)	54.3 (48.4–59.8)	11	56.1 (50.5–61.7)	16
Extraversion (TIPI score)	4 (3–5)	4	4 (3–4.5)	8
Agreeableness (TIPI score)	5.5 (4.5–6.5)	4	5 (4–6)	6
Conscientiousness (TIPI score)	6 (5–6.5)	4	5 (4.5–6.5)	7
Emotional stability (TIPI score)	4.5 (3.5–6)	3	4 (3.5–5.5)	6
Open to new experiences (TIPI score)	5 (4–5.5)	4	4.5 (3.5–5.5)	7
Pain catastrophising (PCS score)	9 (4–17)	2	15.5 (9–30)	6
Traumatic events before 18 years of age (%)	48	3	44	2
Hospital stay before 18 years of age (%)	10	5	11	7
Ever smoked (%)	49	< 1	60	1
Currently drink alcohol (%)	84	< 1	62	< 1
Physical activity (%; high)	54	44	79	20
Health-related quality of life (EQ-5D-5L Index)	0.710 (0.548–0.768)	4	0.567 (0.277–0.710)	6
BMI (kg/m^2^)	27.66 (24.41–31.70)	7	32.68 (29.5525–37.245)	0
Resting heart rate (BPM)	70 (62–77)	4	72 (64.5–81.5)	41
Hypertension (%)	39	7	34	0
Coronary artery disease/heart disease (%)	5	8	29	0
Stroke (%)	2	8	4	0
Glucose (mmol/L)	4.7 (4.4–5.0)	9	8 (6.8–11.375)	46
Creatinine (µmol/L)	70 (63–81)	8	75 (63–93)	1
Total cholesterol (mmol/L)	5.2 (4.5–5.925)	8	4.00 (3.475–4.62)	< 1
High Density Lipoprotein (mmol/L)	1.4 (1.2–1.7)	8	1.00 (0.90–1.31)	1

*BMI* body mass index; *BPM* beats per minute; *EQ-5D-5L* EuroQoL-five dimensions-five levels; *GoDARTS* Genetics of Diabetes Audit and Research In Tayside Scotland; *GS:SFHS* Generation Scotland: Scottish Family Health Study; *PCS* Pain Catastrophising Scale; *PROMIS* patient-reported outcomes measurement information system; *SF4a* short form four answers; *SIMD* Scottish Index of Multiple Deprivation; *TIPI* ten item personality inventory

^a^Observations reported as percentages or median and interquartile range, excluding missing data

### Model development

- Incident neuropathic pain

Once people missing data on neuropathic pain status at follow-up were removed from the analysis (294/4197; Table 2), the total number of people without neuropathic pain at baseline in GS was 3,903. Of these people, 236 (6.0%) had neuropathic pain at follow-up and 3667 did not. For the development of the model (comprising 80% of the GS dataset for the incident neuropathic pain model), there were 189 participants with incident neuropathic pain and 2934 participants who continued to report no neuropathic pain. The remaining 47 participants with incident neuropathic pain and 733 participants with persistent no neuropathic pain were used for internal validation (see Sect. a) Incident neuropathic pain). The predictors with near-zero variance that were removed from further analysis included pain-related worrying, heart rate, CAD/heart disease, stroke, glucose, creatinine and HDL. In the remaining dataset, the proportion of participants with at least one missing predictor observation was 59%.

In the unadjusted univariate analysis conducted on a pooled sample of 59 imputed datasets, depression (odds ratio [OR] = 1.68, 95% confidence interval [CI] = 1.23–2.29), anxiety (OR = 1.38, 95% CI = 1.02–1.87), sleep disturbance (OR = 1.96, 95% CI = 1.44–2.66), emotional stability (OR = 0.71, 95% CI = 0.53–0.97), ACEs (OR = 1.81, 95% CI = 1.34–2.45), past or current smoking (OR = 1.64, 95% CI = 1.22–2.20), HRQoL (OR = 0.29, 95% CI = 0.22–0.40) and BMI (OR = 1.54, 95% CI = 1.06–2.24) were significantly associated with neuropathic pain onset (Supplementary Table S2).

Predictors included in the final multivariate model for incident neuropathic pain were ACEs (*β = *0.369, OR = 1.45, 95% CI = 1.06–1.98), past or current smoking (*β = *0.393, OR = 1.48, 95% CI = 1.10–2.00), HRQoL (*β = *− 1.093, OR = 0.34, 95% CI = 0.24–0.46), being open to new experiences (*β = *0.319, OR = 1.38, 95% CI = 1.01–1.88) and sleep disturbance (*β = *0.421, OR = 1.52, 95% CI = 1.10–2.11) (Table 4). The model had a pooled Nagelkerke *R*^2^ of 0.076 and the likelihood ratio test showed that the model was significantly better at explaining the data compared to an intercept-only model (*P < *0.001).

(b) Resolved neuropathic pain

**Table 4:** Multivariable risk model to predict incident neuropathic pain developed in GS:SFHS

Predictor	Regression coefficient (*β*)	Standard error	Odds ratio (95% confidence interval)	*P* value
Constant	− 2.753	–	–	–
Traumatic events before 18 years of age	0.369	0.159	1.45 (1.06–1.98)	0.019
Ever Smoked	0.393	0.154	1.48 (1.10–2.00)	0.011
Health-related Quality of Life	− 1.093	0.160	0.34 (0.24–0.46)	< 0.001
Open to New Experiences	0.319	0.159	1.38 (1.01–1.88)	0.041
Sleep Disturbance	0.421	0.165	1.52 (1.10–2.11)	0.008

Pooled Nagelkerke *R*^2^ = 0.076

*GS:SFHS* Generation Scotland: Scottish Family Health Study

Once people with missing data on neuropathic pain status at follow-up were removed from the analysis (92/632; Table 3), the total number of people with neuropathic pain at baseline in GS was 540. Of these, 230 people (42.6%) had no neuropathic pain and 310 had neuropathic pain at follow-up. In those with resolved neuropathic pain, 116 people (51%) reported currently taking a medication to treat pain at baseline. For model development there were 184 participants with resolved neuropathic pain and 248 participants with persistent no neuropathic pain (comprising 80% of the GS dataset for the resolved neuropathic pain model). The remaining 46 participants with resolved neuropathic pain and 62 participants with persistent neuropathic pain were used for internal validation (see Sect. b) Resolved neuropathic pain). The predictors with near-zero variance that were removed from further analysis included heart rate, CAD/heart disease, stroke and glucose. In the remaining dataset, the proportion of participants with at least one missing observation was 59%.

In the unadjusted univariate analysis conducted on a pooled sample of 59 imputed datasets, quintile 3 (OR = 2.97, 95% CI = 1.34–6.58), quintile 4 (OR = 2.17, 95% CI = 1.04–4.50) and quintile 5 (OR = 3.24, 95% CI = 1.59–6.57) of the SIMD, depression (OR = 0.50, 95% CI = 0.34–0.75), anxiety (OR = 0.47, 95% CI = 0.31–0.70), sleep disturbance (OR = 0.40, 95% CI = 0.25–0.63), conscientiousness (OR = 3.28, 95% CI = 1.99–5.42), emotional stability (OR = 2.30, 95% CI = 1.55–3.42), pain-related worrying (OR = 0.41, 95% CI = 0.19–0.89), ACEs (OR = 0.63, 95% CI = 0.42–0.93), past or current smoking (OR = 0.64, 95% CI = 0.43–0.94), currently drinking alcohol (OR = 1.87, 95% CI = 1.05–3.34) and HRQoL (OR = 4.69, 95% CI = 2.74–8.04) were significantly associated with resolved neuropathic pain (Supplementary Table S3).

Predictors included in the final multivariate model for resolved neuropathic pain were conscientiousness (*β = *0.807, OR = 2.24, 95% CI = 1.29–3.89), currently drinking alcohol (*β = *0.449, OR = 1.57, 95% CI = 0.83–2.97), emotional stability (*β = *0.324, OR = 1.38, 95% CI = 0.87–2.19), HRQoL (*β = *1.136, OR = 3.11, 95% CI = 1.75–5.55), physical activity (*β = *-0.443, OR = 0.64, 95% CI = 0.35–1.17), sleep disturbance (*β = *-0.529, OR = 0.59, 95% CI = 0.35–1.00) and social deprivation: quintile 2 (*β = *0.545, OR = 1.72, 95% CI = 0.74–4.00), quintile 3 (*β = *0.936, OR = 2.55, 95% CI = 1.08–6.04), quintile 4 (*β = *0.410, OR = 1.51, 95% CI = 0.68–3.33) and quintile 5 (*β = *0.740, OR = 2.10, 95% CI = 0.96–4.57) (Table 5). The model had a pooled Nagelkerke *R*^2^ of 0.219 and the likelihood ratio test showed that the model was significantly better at explaining the data compared to an intercept-only model (*P < *0.001).

**Table 5:** Multivariable risk model to predict resolved neuropathic pain developed in GS:SFHS

Predictor	Regression coefficient (*β*)	Standard error	Odds ratio (95% confidence interval)	*P* value
Constant	− 1.625	–	–	–
Conscientiousness	0.807	0.281	2.24 (1.29–3.89)	0.004
Currently drink alcohol	0.449	0.326	1.57 (0.83–2.97)	0.169
Emotional stability	0.324	0.233	1.38 (0.87–2.19)	0.166
Health-related Quality of Life	1.136	0.294	3.11 (1.75–5.55)	< 0.001
Physical activity	− 0.443	0.304	0.64 (0.35–1.17)	0.147
sleep disturbance	-0.529	0.269	0.59 (0.35–1.00)	0.050
Social deprivation (SIMD)				
1	–	–	–	–
2	0.545	0.428	1.72 (0.74–4.00)	0.204
3	0.936	0.438	2.55 (1.08–6.04)	0.033
4	0.410	0.403	1.51 (0.68–3.33)	0.310
5	0.740	0.396	2.10 (0.96–4.57)	0.062

Pooled Nagelkerke *R*^2^ = 0.219

*GS:SFHS* Generation Scotland: Scottish Family Health Study

### Internal validation

Table 6 provides the full internal validation performance metrics for both the incident neuropathic pain model and the resolved neuropathic pain model.

- Incident neuropathic pain

**Table 6:** Model performance metrics for incident and resolved neuropathic pain

Aspect	Measure	Incident neuropathic pain		Resolved neuropathic pain	
		Internal validation	External validation	Internal validation	External validation
Performance	Nagelkerke *R*^2^	0.091 (0.088–0.093)	0.038 (0.035–0.042)	0.291 (0.277–0.310)	0.148 (0.139–0.161)
Discrimination	AUROC	0.720 (0.716–0.721)	0.636 (0.629–0.643)	0.756 (0.747–0.766)	0.699 (0.695–0.709)
	AUPRC	0.128 (0.126–0.131)	0.152 (0.148–0.157)	0.772 (0.763–0.780)	0.499 (0.484–0.511)
Calibration	Slope	1.033 (1.019–1.053)	0.623 (0.592–0.658)	1.227 (1.177–1.287)	0.999 (0.962–1.041)
	Intercept	0.040 (‒0.007, 0.092)	‒0.511 (‒0.593, ‒0.426)	0.184 (0.158–0.215)	‒0.424 (‒0.454, ‒0.394)
Clinical Utility	Net benefit at incidence threshold^a^	n/a	0.022 (0.020–0.022)^b^	n/a	0.065 (0.061–0.070)^b^

All measures presented as the median and interquartile range

*AUPRC* area under the precision-recall curve; *AUROC* area under the receiver operating characteristic; *GoDARTS* Genetics of Diabetes Audit and Research in Tayside Scotland

^a^Incidence threshold (GoDARTS): Incident Neuropathic pai*n = *10.7%, Resolved Neuropathic Pai*n = *23.7%

^b^Adjusted for calibration

Internal validation in GS consisted of the 47 participants with incident neuropathic pain and 733 participants with persistent no neuropathic pain (comprising 20% of the GS dataset for the incident neuropathic pain model). The model discriminated well in terms of the ROC curve, with an AUC of 0.720, whilst the AUPRC was 0.128. The calibration performance was also good, with a median slope and intercept of 1.033 and 0.040 respectively. The median additional fraction of the variance in incident neuropathic pain explained by the developed model over the null model according to the Nagelkerke *R*^2^ was 0.091 (Table 6).

A breakdown of the incident neuropathic pain model internal validation performance metrics in each imputed dataset is provided in Supplementary Table S4 and example plots of the performance metrics for the ROC, PRC and calibration curves in dataset 32 are given in Supplementary Figures S1–S3.

(b) Resolved neuropathic pain

There were 46 participants with resolved neuropathic pain and 62 participants with persistent neuropathic pain in the GS internal validation dataset (comprising 20% of the GS dataset for the resolved neuropathic pain model). The model showed good discrimination in both the ROC curve and PRC with a median AUC of 0.756 and 0.772 respectively. The calibration curve showed evidence of model underfitting with a median slope of 1.227 and median intercept of 0.184 respectively. The median additional fraction of the variance in resolved neuropathic pain explained by the developed model over the null model according to the Nagelkerke *R*^2^ was 0.291 (Table 6).

A breakdown of the resolved neuropathic pain model internal validation performance metrics in each imputed dataset is provided in Supplementary Table S6 and example plots of the performance metrics for the ROC, PRC and calibration curves in dataset 29 are given in Supplementary Figures S4–S6.

### External validation

Table 6 provides the full external validation performance metrics for both the incident neuropathic pain model and the resolved neuropathic pain model.

- Incident neuropathic pain

**Fig. 1:**
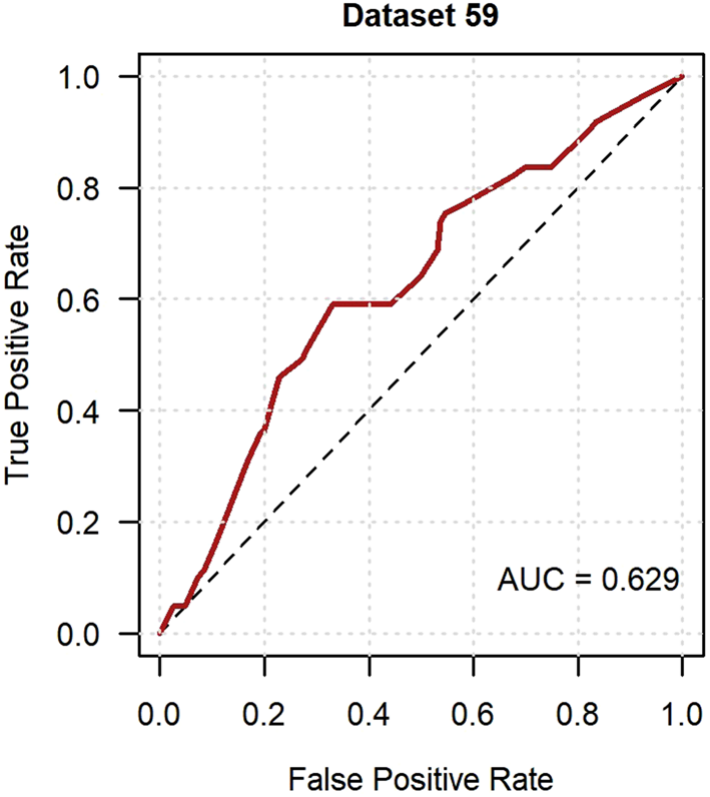
Receiver operating characteristic curve of the incident neuropathic pain model in GoDARTS. Imputed dataset 59 was randomly chosen to represent model discrimination and the AUC was 0.629

**Fig. 2:**
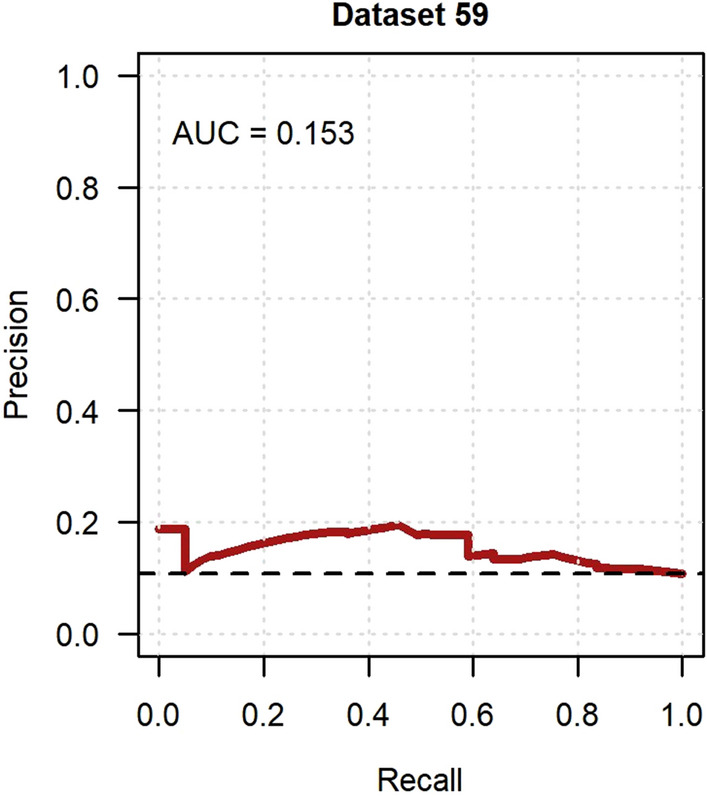
Precision-recall curve of the incident neuropathic pain model in GoDARTS. Imputed dataset 59 was randomly chosen to represent model discrimination and the AUC was 0.153

**Fig. 3:**
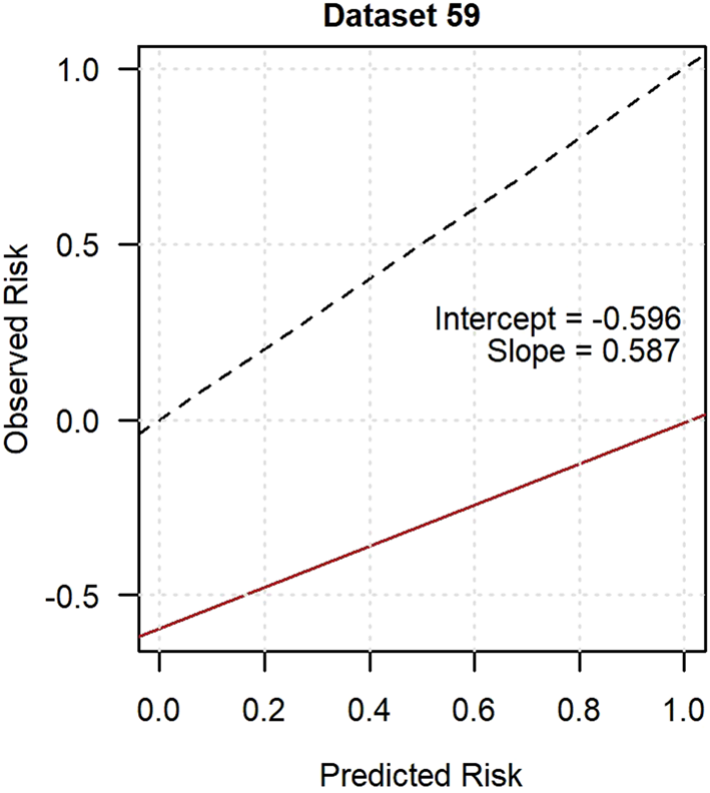
Calibration plot of the incident neuropathic pain model in GoDARTS. Imputed dataset 59 was randomly chosen to represent model calibration and the intercept was − 0.596 and the slope was 0.587

**Fig. 4:**
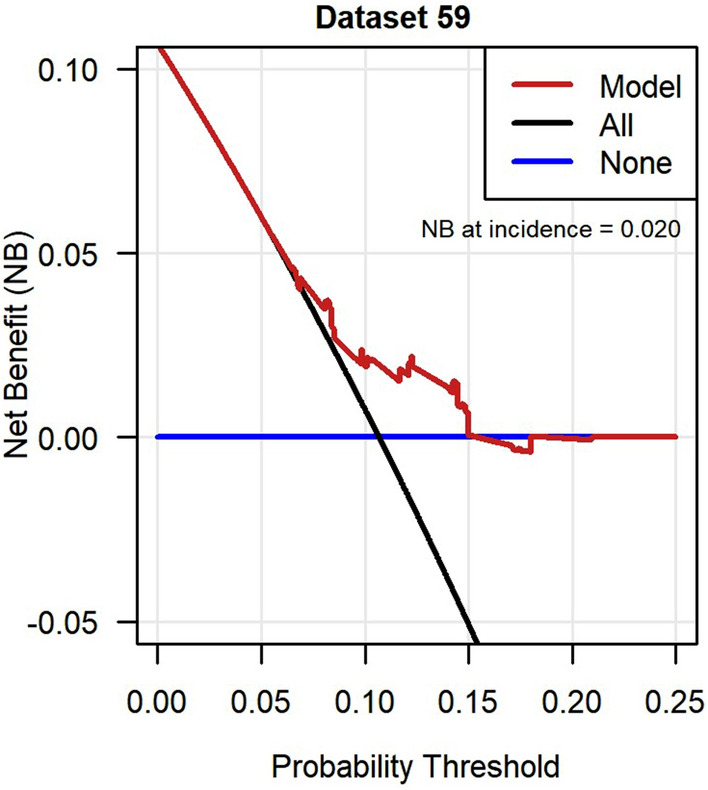
Decision curve analysis of net benefit of the incident neuropathic pain model in GoDARTS. Imputed dataset 59 was randomly chosen to represent clinical utility and the net benefit at outcome incidence (10.7%) was 0.020

External validation in GoDARTS was conducted on 61 participants with incident neuropathic pain (10.7%) and 510 participants with persistent no neuropathic pain. Imputed dataset 59 was randomly chosen to visually demonstrate the performance measures. The model showed acceptable discrimination in the ROC curve analysis with a median AUC of 0.636 (Fig. 1), whilst the median AUPRC was 0.152 (Fig. 2). The calibration curve showed evidence of model overfitting with a median slope of 0.623 and median intercept of − 0.511 (Fig. 3). The median additional fraction of the variance in incident neuropathic pain explained by the developed model over the null model according to the Nagelkerke *R*^2^ was 0.038. Figure 4 shows the result of the DCA in dataset 59. The model was comparable to a “treat all” approach up to a threshold probability of 7%. Between 7 and 15% the model had a higher net benefit than both the “treat all” and “treat none” approach. Above 15% the model was either comparable or slightly worse than the “treat none” approach. The median net benefit at the incidence of neuropathic pain threshold (10.7%) across all imputed datasets was 0.022. This is equivalent to 22 patients being correctly treated per 1,000. Compared to the “treat all” and “treat none” approaches this is the net benefit for no increase in the number of patients being incorrectly treated.

A breakdown of the external validation performance metrics for the incident neuropathic pain model in each imputed dataset is provided in Supplementary Table S5.

(b) Resolved neuropathic pain

**Fig. 5:**
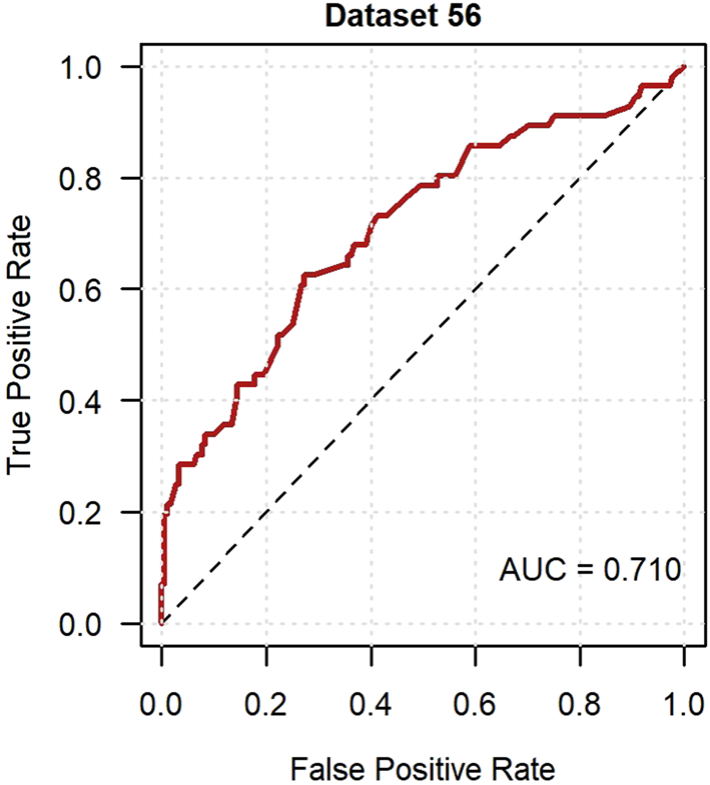
Receiver operating characteristic curve of the resolved neuropathic pain model in GoDARTS. Imputed dataset 56 was randomly chosen to represent model discrimination and the AUC was 0.710

**Fig. 6:**
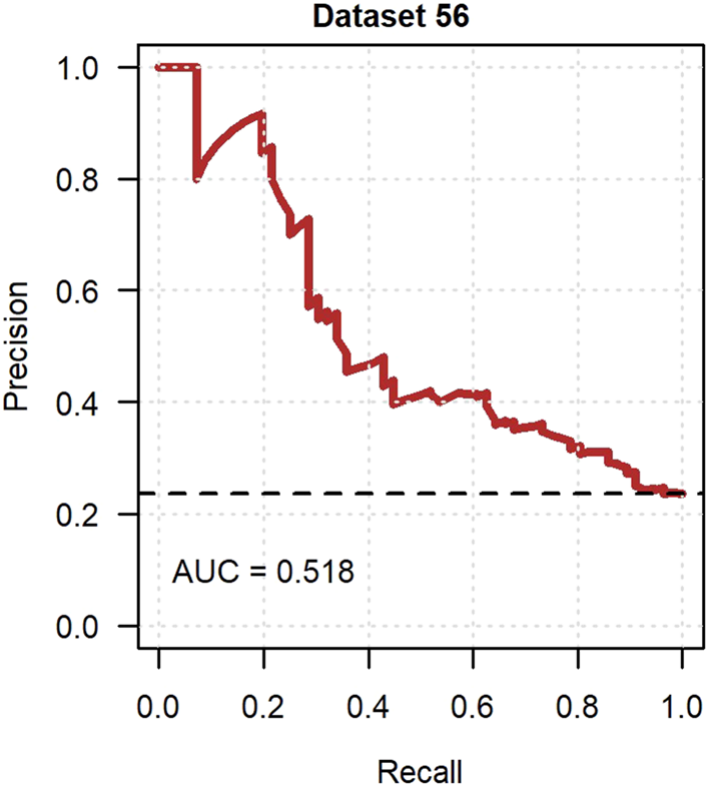
Precision-recall curve of the resolved neuropathic pain model in GoDARTS. Imputed dataset 56 was randomly chosen to represent model discrimination and the AUC was 0.518

**Fig. 7:**
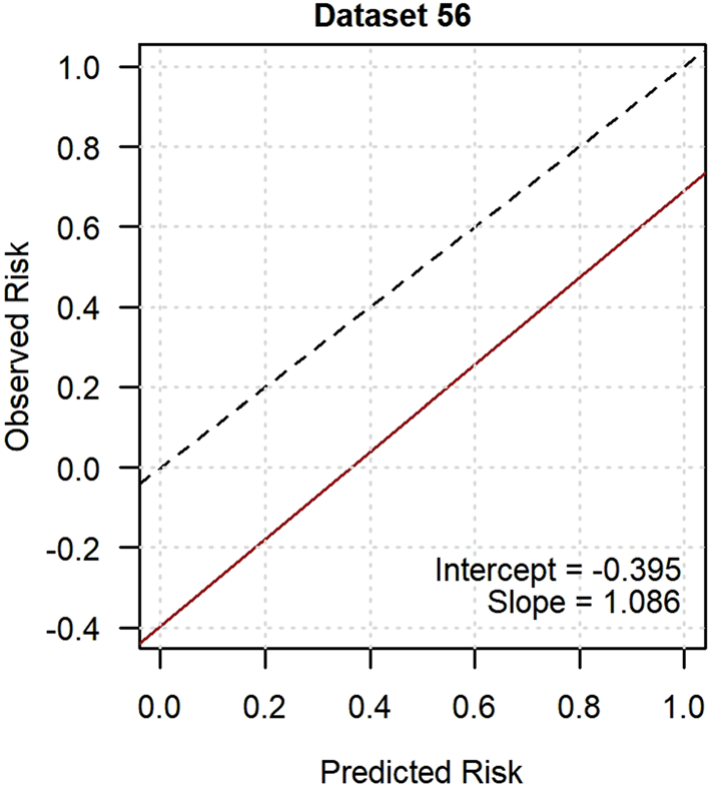
Calibration plot of the resolved neuropathic pain model in GoDARTS. Imputed dataset 56 was randomly chosen to represent model calibration and the intercept was − 0.395 and the slope was 1.086

External validation in GoDARTS was conducted on 56 participants with resolved neuropathic pain (23.7%) and 180 participants with persistent neuropathic pain. In those with resolved neuropathic pain, 33 people (59%) reported currently taking a medication to treat pain at baseline. Imputed dataset 56 was randomly chosen to visually demonstrate the performance measures. The model showed near-good discrimination in the ROC curve analysis with a median AUC of 0.699 (Fig. 5), whilst the median AUPRC was 0.499 (Fig. 6). The calibration curve had a near-ideal median slope of 0.999, however the median intercept of -0.424 suggested overfitting (Fig. 7). The median additional fraction of the variance in resolved neuropathic pain explained by the developed model over the null model according to the Nagelkerke *R*^2^ was 0.148.

**Fig. 8:**
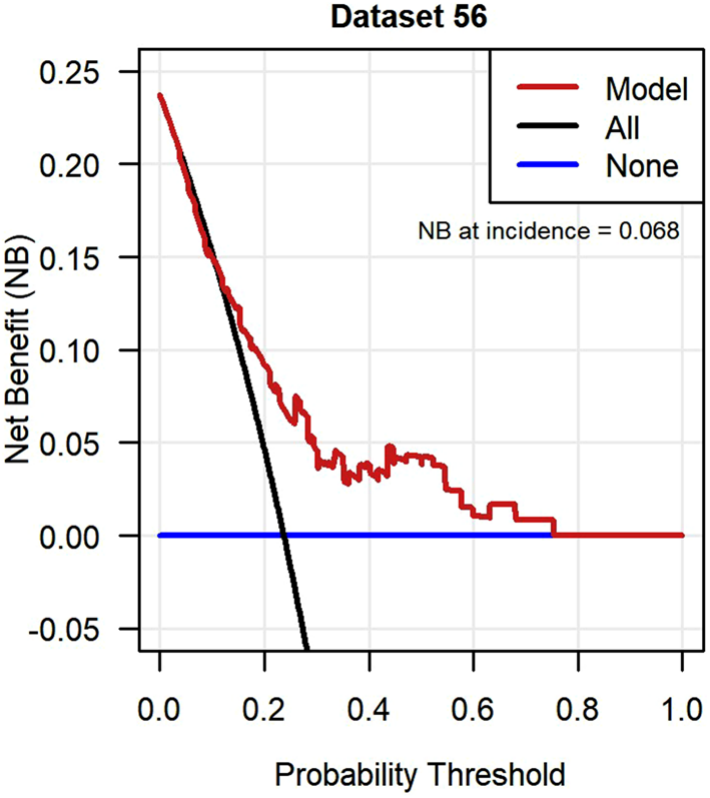
Decision curve analysis of net benefit of the incident neuropathic pain model in GoDARTS. Imputed dataset 56 was randomly chosen to represent clinical utility and the net benefit at outcome incidence (23.7%) was 0.068

Figure 8 shows the result of the DCA in dataset 56. The model was comparable or slightly worse than the “treat all” approach up to a threshold probability of 12%. Between 12 and 72% the model had a higher net benefit than both the “treat all” and “treat none” approach. Above 72% the model was comparable to the “treat none” approach. The median net benefit at the incidence of resolved neuropathic pain (23.7%) across all imputed datasets was 0.065. This is equivalent to 65 patients being correctly treated per 1,000 for no increase in the number of patients being incorrectly treated, compared to the “treat all” and “treat none” approaches.

A breakdown of the external validation performance metrics for the resolved neuropathic pain model in each imputed dataset is provided in Supplementary Table S7.

### Model algorithms

A risk score for incident neuropathic pain, corrected for calibration in the validation cohort, can be derived from the following algorithm:

Risk score = − 0.511 + 0.623 × (− 2.753 + [0.369 × Adverse Childhood Experiences] + [0.393 × Ever Smoked] + [− 1.093 × Health-related Quality of Life] + [0.319 × Open to New Experiences] + [0.421 × Sleep Disturbance]).

The corresponding calibration corrected risk score for resolved neuropathic pain can be calculated using the following algorithm:

Risk score = -− 0.424 + 0.999 × (− 1.625 + [0.807 × Conscientiousness] + [0.449 × Currently Drink Alcohol] + [0.324 × Emotional Stability] + [1.136 × Health-related Quality of Life] + [− 0.443 × Physical Activity] + [− 0.529 × Sleep Disturbance] + [0.545 × Social Deprivation 2] + [0.936 × Social Deprivation 3] + [0.410 × Social Deprivation 4] + [0.740 × Social Deprivation 5]).

Where Adverse Childhood Experiences = 0 (no traumatic events before 18 years) or 1 (at least 1 traumatic event before 18 years), Conscientiousness = 0 (TIPI < 5.0) or 1 (TIPI ≥ 5.0), Currently Drink Alcohol = 0 (no) or 1 (yes), Emotional Stability = 0 (TIPI < 5.0) or 1 (TIPI ≥ 5.0), Ever Smoked = 0 (never) or 1 (current or past), Health-related Quality of Life = 0 (EQ-5D-5L < 0.800) or 1 (EQ-5D-5L ≥ 0.800), Open to New Experiences = 0 (TIPI < 5.0) or 1 (TIPI ≥ 5.0), Physical Activity = 0 (Low) or 1 (High), Sleep Disturbance = 0 (PROMIS *T*-score < 50) or 1 (PROMIS *T*-score ≥ 50), Sleep Disturbance = 0 (PROMIS *T*-score < 50) or 1 (PROMIS *T*-score ≥ 50), Social Deprivation 2 = 0 (SIMD Quintile 1/3/4/5) or 1 (SIMD Quintile 2), Social Deprivation 3 = 0 (SIMD Quintile 1/2/4/5) or 1 (SIMD Quntile 3), Social Deprivation 4 = 0 (SIMD Quintile 1/2/3/5) or 1 (SIMD Quintile 4), Social Deprivation 5 = 0 (SIMD Quintile 1/2/3/4) or 1 (SIMD Quintile 5).

To obtain probabilities for either outcome (on a 0 to 1 scale), the following formula should be used:

$${\text{Outcome probability}} = {\text{exp}}\left( {\text{Risk score}} \right)/\left( {{1}\, + \,{\text{exp}}\left[ {\text{Risk score}} \right]} \right).$$

## Discussion

### Summary

To the best of our knowledge this is the first study to report externally validated risk models for neuropathic pain outcomes. Both models performed well in general Scottish population samples, with good discrimination and calibration metrics, but in a higher risk cohort of predominantly type 2 diabetics, there was some evidence that the models had been overfitted to the development dataset. The DCA results indicate that the models could have clinical utility in mitigating the risks of incident and refractory neuropathic pain in community-based settings.

### Strengths and limitations

A major strength of this study is the longitudinal design. This is one key factor for inferring the causal relationship between potential predictors and neuropathic pain, many of which could feasibly be bidirectional [52]. To date, only a few longitudinal studies have been conducted to investigate predictors of neuropathic pain onset [52–55]. Despite the current study having low absolute case numbers, which may limit statistical power, compared to these previous studies, it has the largest sample size in the development cohort (GS; *n = *3,903), was the first to be conducted in general and diabetic populations and was the first to consider neuropathic pain resolution. Although focussed on risk prediction, the study also revealed incidences of neuropathic pain onset (6.0% in GS and 10.7% in GoDARTS) and resolution (42.6% in GS and 23.7% in GoDARTS). It will be interesting to compare these estimates in general and diabetic cohorts from different populations in future studies.

A particular strength of this study was the validation of the model in an independent cohort. External validation demonstrates the generalisability and reproducibility of the models in different populations, potentially with differences in causative mechanisms. This was possible using the same questionnaires in the development and validation cohorts, meaning that there were no differences in the outcome phenotype nor the predictor variables, thereby reducing the risk of introducing bias into the models.

Another strength is the way the data were collected and analysed. Many of the variables analysed in this study were part of a “core” phenotype implemented in DOLORisk [17] and closely followed in other cohorts [56]. Further planned replication and validation of these findings in independent cohorts, both inside and outside the consortium, should be straightforward as they become available. The predictors included in the final models are potentially more amenable to use in routine clinical settings than more complex neurological and sensory tests (e.g. quantitative sensory testing) that are used in specialist clinics. Their interpretation has been aided by their dichotomisation into binary categories.

This study also has some limitations. First, our definition of neuropathic pain did not specify pain location, cause, or intensity. Therefore, it is possible that the nature of the pain reported by participants may have changed between baseline and follow-up. Hence, we could not explore individual aetiologies of neuropathic pain nor compare it to participants with no pain or nociceptive pain individually. Second, no clinical examination to reveal sensory signs in the body region affected by neuropathic pain was possible, which forms part of the grading system for neuropathic pain [57]. Instead, we used the DN4 to screen for neuropathic pain, which has 78% sensitivity and 81% specificity when used as a self-report questionnaire [29]. Third, the response rates in the baseline survey were low (~ 36% in both cohorts). A low response rate can lead to sampling bias if it creates a cohort that is not representative of the population that it has been drawn from. However, we have previously demonstrated that demographic characteristics in both GS and GoDARTS are clinically similar between respondents to the baseline DOLORisk Dundee questionnaire and the overall cohorts [18]. Demographic characteristics were also similar between respondents and non-respondents to the follow-up questionnaire. Moreover, it has been reported that studies with low response rates can have the same validity as those with good response rates [58–61]. It should be noted that the response rates reported in this study are similar to those in previous studies of this nature [62, 63].

### Interpretation

Our findings do provide a valuable starting point for developing a tool which can be used to predict incident or resolved neuropathic pain in a clinical setting. The factors identified in the models also reveal important information on the potential pathways and mechanisms that are involved in the onset and progression of neuropathic pain and this provides insights into potential areas for developing treatments and prevention strategies. Comparisons can also be made to risk models developed in other pain types, some of which may have neuropathic components, despite not being screened for specifically [64, 65].

Both the incident and resolved neuropathic pain models had comparable discrimination to studies in other pain phenotypes, though none of these studies reported on precision and recall [64–66]. In this study, both models demonstrated better than chance discrimination through their precision and recall metrics. Despite this, the calibration curves indicate that the probabilities produced by both models are overestimated. This is supported by the reduction in median Nagelkerke *R*^2^ between internal and external validation. Together this suggests reduced generalisability of the models in GoDARTS. A possible reason for this could be the underlying differences between GoDARTS and GS in terms of the clinical features, demographics and model predictors [18].

Clinical utility is not widely reported for prediction models [45], though there is a notable exception in acute low back pain [64]. The clinical utility of a model is ultimately decided by patients and clinicians by how they weigh up the potential benefit and harms of treatment. For example treatment could involve physical activity, where there is low risk of harm [67]. For this low-risk intervention a low probability threshold for treatment might be appropriate, implying that correctly treating a true positive case, outweighs unnecessarily treating a false-positive. Conversely, interventions involving medications, such as gabapentinoids, or surgical interventions may carry greater risk of harm and a higher threshold considered. Therefore, a model that is beneficial over a wide range of probabilities compared to a “treat all” or “treat none” approach is preferable.

Alcohol consumption, smoking and physical activity are examples of predictors that may be amenable to modification and, therefore, targeted by interventions. It is interesting to note the apparent protective effect of being a current drinker of alcohol in relation to resolved neuropathic pain, considering that heavy drinkers can develop alcohol-induced neuropathy and it increases risk of painful diabetic neuropathy [68, 69]. Conversely, the risk of being a current or past smoker with incident neuropathic pain is in line with a previous finding in postherpetic neuralgia [55].

The strongest predictor in both models was HRQoL, assessed using the EQ5D-5L. The higher the HRQoL, the lower the likelihood of neuropathic pain onset and the greater the chances of neuropathic pain resolution. In previous cross-sectional studies, HRQoL was consistently lower in participants with neuropathic pain [3, 9, 70, 71]. Furthermore, one longitudinal study reported a protective effect of the physical component summary of the 12-item short-form health survey with postherpetic neuralgia [54]. Physical activity has also been associated with risk of neuropathic pain in survivors of myocardial infarction with diabetes, in line with the protective association it has in resolving neuropathic pain in this study [72].

The inclusion of HRQoL at the expense of other constructs in both models is perhaps unsurprising given the multiple domains that the EQ5D-5L covers including anxiety and depression [22]. Although none of the questionnaires assessing these domains separately were included in the final models, they are frequently reported comorbidities with neuropathic pain [53, 73] and genetic studies have suggested shared heritability with pain in general [28, 74]. It is possible that the inclusion of HRQoL as a strong predictor may act as a summary proxy for these multiple domains. Depression and anxiety are also likely to be related to the personality items that were included in the models such as emotional stability.

Being open to new experiences and childhood trauma are both novel findings that were associated with a higher likelihood of neuropathic pain onset, though the latter has been associated with the development of chronic pain in adult life [75]. Both factors require further exploration to better understand the mechanisms involved.

Sleep disturbance is another factor that was included in both models and this is supported by a previous longitudinal study that reported a bidirectional relationship with neuropathic pain following total joint replacement [52]. Another study of 16 pooled clinical trials of painful diabetic peripheral neuropathy and postherpetic neuralgia found that severe sleep disturbance was predictive of pain relief in individuals taking pregabalin [76].

### Further work

Because the models described in this study were developed in the general population, they are applicable for estimating risk of incident or resolved neuropathic pain in patients in the community, presenting in a non-specialist setting such as primary care. Future work should concentrate on replicating these models further, including more intensive phenotyping, to improve their validity and generalisability. Further analysis should also be conducted in individual aetiologies of neuropathic pain.

Although genetic factors have not been analysed in this study, they have been studied separately elsewhere in DOLORisk Dundee [77]. These two components should be brought together to analyse the relative contribution of genetic and environmental factors to neuropathic pain. Furthermore, Mendelian randomisation analysis can be used to establish the causal effects of the predictors identified in this study.

In participants who had resolved neuropathic pain, it was not possible to establish definitively whether this was spontaneous or due to treatment. However, data from the baseline survey suggest around half of this group in GS and GoDARTS were taking medications to treat pain. Future studies will focus on linking the DOLORisk Dundee questionnaire data to routinely collected NHS prescribing data, to allow analysis of the analgesic medications that people with neuropathic pain are taking, whether this conforms to clinical guidelines, and how it is related to clinical outcomes. The risk model for resolved neuropathic pain can also be further developed by analysing how the predictors affect patient response to specific neuropathic pain medications, such as gabapentinoids, tricyclic antidepressants (TCAs) and Serotonin-Norepinephrine reuptake inhibitors (SNRIs).

## Conclusion

This study quantifies the role of psychological and lifestyle risk factors in the development and resolution of neuropathic pain. These findings will be of value both to healthcare professionals who will be able to raise awareness in people at high risk, and to patients who potentially could attempt preventative measures for modifiable factors. The findings require further investigation and updating to improve their applicability to different neuropathic pain populations.

## Supplementary Information

Below is the link to the electronic supplementary material.

Supplementary file1 (DOCX 472 kb)

## Data Availability

DOLORisk Dundee data will be deposited in the Alleviate Data Hub, part of the Advanced Pain Discovery Platform. Details on how to access the data will be made available through the Alleviate website (https://alleviate.ac.uk/).
