# Correction: Development and external validation of multivariable risk models to predict incident and resolved neuropathic pain: a DOLORisk Dundee study

**DOI:** 10.1007/s00415-024-12721-6

**Published:** 2024-11-13

**Authors:** Harry L. Hébert, Abirami Veluchamy, Georgios Baskozos, Francesca Fardo, Dimitri Van Ryckeghem, Ewan R. Pearson, Lesley A. Colvin, Geert Crombez, David L. H. Bennett, Weihua Meng, Colin N. A. Palmer, Blair H. Smith

**Affiliations:** 1 Chronic Pain Research Group, Division of Population Health and Genomics, Mackenzie Building, Ninewells Hospital and Medical School, University of Dundee, Kirsty Semple Way, Dundee, DD2 4BF UK; 2 Pat Macpherson Centre for Pharmacogenetics and Pharmacogenomics, Division of Population Health and Genomics, Ninewells Hospital and Medical School, University of Dundee, Dundee, UK; 3 Neural Injury Group, Nuffield Department of Clinical Neuroscience, John Radcliffe Hospital, University of Oxford, Oxford, UK; 4 Danish Pain Research Center, Department of Clinical Medicine, Aarhus University, Aarhus, Denmark; 5 Department of Experimental-Clinical and Health Psychology, Faculty of Psychology and Educational Sciences, Ghent University, Ghent, Belgium; 6 Section Experimental Health Psychology, Clinical Psychological Science, Departments, Faculty of Psychology and Neuroscience, Maastricht University, Maastricht, Netherlands

**Correction: Journal of Neurology (2022) 270:1076-1094** 10.1007/s00415-022-11478-0

In the original version of this article, the funding information section was incorrectly given as

This work was supported by the European Union’s Horizon 2020 research and innovation programme under grant agreement No 633491 (DOLORisk). BHS, CNAP, DLHB, GC and WM are members of the DOLORisk consortium and are partly supported by this grant. HLH and AV are supported by DOLORisk. Generation Scotland received core support from the Chief Scientist Office of the Scottish Government Health Directorates [CZD/16/6] and the Scottish Funding Council [HR03006]. The Wellcome Trust United Kingdom Type 2 Diabetes Case–Control Collection (supporting GoDARTS) was funded by The Wellcome Trust (072960/Z/03/Z, 084726/Z/08/Z, 084727/Z/08/Z, 085475/Z/08/Z, and 085475/B/08/Z) and as part of the EU IMI-SUMMIT program. This research was also partly supported by the NIHR Oxford Health Biomedical Research Centre (NIHR203316), and by the Wellcome Trust (203139/Z/16/Z and 203139/A/16/Z).

and should have read

This work was supported by the European Union’s Horizon 2020 research and innovation programme under grant agreement No 633491 (DOLORisk). BHS, CNAP, DLHB, GC and WM are members of the DOLORisk consortium and are partly supported by this grant. HLH and AV are supported by DOLORisk. Generation Scotland received core support from the Chief Scientist Office of the Scottish Government Health Directorates [CZD/16/6] and the Scottish Funding Council [HR03006]. The Wellcome Trust United Kingdom Type 2 Diabetes Case–Control Collection (supporting GoDARTS) was funded by The Wellcome Trust (072960/Z/03/Z, 084726/Z/08/Z, 084727/Z/08/Z, 085475/Z/08/Z, and 085475/B/08/Z) and as part of the EU IMI-SUMMIT program. This research was also partly supported by the NIHR Oxford Health Biomedical Research Centre (NIHR203316), and by the Wellcome Trust (203139/Z/16/Z and 203139/A/16/Z). FF was supported by a European Research Council Starting Grant (ERC-2020-StG-948838).

