# Supplementary material for: Development and external validation of multivariable risk models to predict incident and resolved neuropathic pain: a DOLORisk Dundee study
Source: J Neurol. 2022 Nov 10;270(2):1076–94. doi: 10.1007/s00415-022-11478-0 (PMC9886655; doi:10.1007/s00415-022-11478-0)
Supplement: Supplementary file 1 — Supplementary file1 (DOCX 472 kb) [file 415_2022_11478_MOESM1_ESM.docx]

Supplemental Digital Content

**Development and external validation of multivariable risk models to predict incident and resolved neuropathic pain in the Scottish population: a DOLORisk Dundee study.**

Harry L Hébert^1^, Abirami Veluchamy^1,2^, Georgios Baskozos^3^, Francesca Fardo^4^,

Dimitri Van Ryckeghem^5,6^, Ewan R Pearson^2^, Lesley A. Colvin^1^, Geert Crombez^5^, David L H Bennett^3^, Weihua Meng^1^, Colin N A Palmer^2^, Blair H Smith^1^*

^1^Chronic Pain Research Group, Division of Population Health and Genomics, Mackenzie Building, Ninewells Hospital & Medical School, University of Dundee, Dundee, UK

^2^Pat Macpherson Centre for Pharmacogenetics and Pharmacogenomics, Division of Population Health and Genomics, Ninewells Hospital & Medical School, University of Dundee, Dundee, UK

^3^Neural Injury Group, Nuffield Department of Clinical Neuroscience, John Radcliffe Hospital, University of Oxford, Oxford, UK

^4^Danish Pain Research Center, Department of Clinical Medicine, Aarhus University, Denmark

^5^Department of Experimental-Clinical and Health Psychology, Faculty of Psychology and Educational Sciences, Ghent University, Ghent, Belgium

^6^Section Experimental Health Psychology, Clinical Psychological Science, Departments, Faculty of Psychology and Neuroscience, Maastricht University, Netherlands

Contents

[**Supplementary Table S1.** TRIPOD Checklist: Prediction Model Development and Validation](#_Toc78476487)

[**Supplementary Table S2** – Univariate analysis of incident neuropathic pain in GS:SFHS (pooled analysis after multiple imputation m = 59)](#_Toc78476488)

[**Supplementary Table S3** - Univariate analysis of resolved neuropathic pain in GS:SFHS (pooled analysis after multiple imputation m = 59)](#_Toc78476489)

[**Supplementary Table S4.** Internal validation performance metrics in each imputed dataset for the risk model for incident neuropathic pain](#_Toc78476490)

[**Supplementary Table S5.** External validation performance metrics in each imputed dataset for the risk model for incident neuropathic pain](#_Toc78476491)

[**Supplementary Table S6.** Internal validation performance metrics in each imputed dataset for the risk model for resolved neuropathic pain](#_Toc78476492)

[**Supplementary Table S7.** External validation performance metrics in each imputed dataset for the risk model for resolved neuropathic pain](#_Toc78476493)

[**Supplementary Figure S1.** ROC curve for internal validation of the risk model for incident neuropathic pain (imputed dataset 32)](#_Toc78476494)

[**Supplementary Figure S2.** Precision-recall curve for internal validation of the risk model for incident neuropathic pain (imputed dataset 32)](#_Toc78476495)

[**Supplementary Figure S3.** Calibration curve for internal validation of the risk model for incident neuropathic pain (imputed dataset 32)](#_Toc78476496)

[**Supplementary Figure S4.** ROC curve for internal validation of the risk model for resolved neuropathic pain (imputed dataset 29)](#_Toc78476497)

[**Supplementary Figure S5.** Precision-recall curve for internal validation of the risk model for resolved neuropathic pain (imputed dataset 29)](#_Toc78476498)

[**Supplementary Figure S6.** Calibration curve for internal validation of the risk model for resolved neuropathic pain (imputed dataset 29)](#_Toc78476499)

# **Supplementary Table S1.** TRIPOD Checklist: Prediction Model Development and Validation

| **Section/Topic** | **Item** |  | **Checklist Item** | **Page** |
| --- | --- | --- | --- | --- |
| **Title and abstract** | | | | |
| Title | 1 | D;V | Identify the study as developing and/or validating a multivariable prediction model, the target population, and the outcome to be predicted. | Title Page |
| Abstract | 2 | D;V | Provide a summary of objectives, study design, setting, participants, sample size, predictors, outcome, statistical analysis, results, and conclusions. | Abstract |
| **Introduction** | | | | |
| Background and objectives | 3a | D;V | Explain the medical context (including whether diagnostic or prognostic) and rationale for developing or validating the multivariable prediction model, including references to existing models. | Introduction  Paragraphs 1-4 |
|  | 3b | D;V | Specify the objectives, including whether the study describes the development or validation of the model or both. | Introduction  Paragraph 5 |
| **Methods** | | | | |
| Source of data | 4a | D;V | Describe the study design or source of data (e.g., randomized trial, cohort, or registry data), separately for the development and validation data sets, if applicable. | Methods  Study Design Paragraphs 1-3 |
|  | 4b | D;V | Specify the key study dates, including start of accrual; end of accrual; and, if applicable, end of follow-up. | Methods  Recruitment Paragraphs 1-2 |
| Participants | 5a | D;V | Specify key elements of the study setting (e.g., primary care, secondary care, general population) including number and location of centres. | Methods  Study Design Paragraphs 1-3 |
|  | 5b | D;V | Describe eligibility criteria for participants. | Methods  Recruitment Paragraphs 1-2 |
|  | 5c | D;V | Give details of treatments received, if relevant. | n/a |
| Outcome | 6a | D;V | Clearly define the outcome that is predicted by the prediction model, including how and when assessed. | Methods  Outcome |
|  | 6b | D;V | Report any actions to blind assessment of the outcome to be predicted. | n/a |
| Predictors | 7a | D;V | Clearly define all predictors used in developing or validating the multivariable prediction model, including how and when they were measured. | Methods  Predictors  Table 1 |
|  | 7b | D;V | Report any actions to blind assessment of predictors for the outcome and other predictors. | n/a |
| Sample size | 8 | D;V | Explain how the study size was arrived at. | Methods  Sample Size  Paragraphs 1-2 |
| Missing data | 9 | D;V | Describe how missing data were handled (e.g., complete-case analysis, single imputation, multiple imputation) with details of any imputation method. | Methods  Missing Data  Paragraphs 1-2 |
| Statistical analysis methods | 10a | D | Describe how predictors were handled in the analyses. | Methods  Statistical Analysis  Paragraph 2 |
|  | 10b | D | Specify type of model, all model-building procedures (including any predictor selection), and method for internal validation. | Methods  Statistical Analysis  Paragraph 2 and 5 |
|  | 10c | V | For validation, describe how the predictions were calculated. | Methods  Statistical Analysis  Paragraph 5 |
|  | 10d | D;V | Specify all measures used to assess model performance and, if relevant, to compare multiple models. | Methods  Statistical Analysis  Paragraphs 3-8 |
|  | 10e | V | Describe any model updating (e.g., recalibration) arising from the validation, if done. | Methods  Statistical Analysis  Paragraph 7 |
| Risk groups | 11 | D;V | Provide details on how risk groups were created, if done. | n/a |
| Development vs. validation | 12 | V | For validation, identify any differences from the development data in setting, eligibility criteria, outcome, and predictors. | Methods  Study Design  Paragraph 3 |
| **Results** | | | | |
| Participants | 13a | D;V | Describe the flow of participants through the study, including the number of participants with and without the outcome and, if applicable, a summary of the follow-up time. A diagram may be helpful. | Results  Participant Flow  Paragraph 1 |
|  | 13b | D;V | Describe the characteristics of the participants (basic demographics, clinical features, available predictors), including the number of participants with missing data for predictors and outcome. | Results  Baseline Characteristics  Tables 2-3 |
|  | 13c | V | For validation, show a comparison with the development data of the distribution of important variables (demographics, predictors and outcome). | Results  Baseline Characteristics  Tables 2-3 |
| Model development | 14a | D | Specify the number of participants and outcome events in each analysis. | Results  Model Development  Paragraphs 1 and 4 |
|  | 14b | D | If done, report the unadjusted association between each candidate predictor and outcome. | Results  Model Development  Paragraphs 2 and 5  Supplementary Tables S1-S2 |
| Model specification | 15a | D | Present the full prediction model to allow predictions for individuals (i.e., all regression coefficients, and model intercept or baseline survival at a given time point). | Results  Model Development  Paragraphs 3 and 6  Tables 4-5 |
|  | 15b | D | Explain how to the use the prediction model. | Results  Model Algorithms |
| Model performance | 16 | D;V | Report performance measures (with CIs) for the prediction model. | Results  Model Development  Table 6 |
| Model-updating | 17 | V | If done, report the results from any model updating (i.e., model specification, model performance). | Results  Model Algorithms |
| **Discussion** | | | | |
| Limitations | 18 | D;V | Discuss any limitations of the study (such as nonrepresentative sample, few events per predictor, missing data). | Discussion  Strengths and Limitations  Paragraph 4 |
| Interpretation | 19a | V | For validation, discuss the results with reference to performance in the development data, and any other validation data. | Discussion  Interpretation  Paragraphs 2-3 |
|  | 19b | D;V | Give an overall interpretation of the results, considering objectives, limitations, results from similar studies, and other relevant evidence. | Discussion  Interpretation  Paragraphs 4-8 |
| Implications | 20 | D;V | Discuss the potential clinical use of the model and implications for future research. | Discussion  Further Work  Paragraphs 1-3 |
| **Other information** | | | | |
| Supplementary information | 21 | D;V | Provide information about the availability of supplementary resources, such as study protocol, Web calculator, and data sets. | Methods  Study Design  Paragraph 1 |
| Funding | 22 | D;V | Give the source of funding and the role of the funders for the present study. | Acknowledgements  Paragraphs 1-3 |

*Items relevant only to the development of a prediction model are denoted by D, items relating solely to a validation of a prediction model are denoted by V, and items relating to both are denoted D;V. We recommend using the TRIPOD Checklist in conjunction with the TRIPOD Explanation and Elaboration document.

# **Supplementary Table S2** – Univariate analysis of incident neuropathic pain in GS:SFHS (pooled analysis after multiple imputation m = 59)

| **Characteristic** | **Odds Ratio (95% Confidence Interval)** | **Standard Error** | **P-value** |
| --- | --- | --- | --- |
| Age (≥65 years) | 1.07 (0.79-1.46) | 0.158 | 0.654 |
| Gender (Male) | 1.11 (0.83-1.50) | 0.152 | 0.476 |
| Social Deprivation (SIMD)  1 (Most deprived)  2  3  4  5 (Least deprived) | -  0.93 (0.45-1.91)  0.73 (0.37-1.46)  0.86 (0.46-1.61)  0.55 (0.29-1.03) | -  0.370  0.353  0.320  0.322 | -  0.835  0.378  0.638  0.062 |
| Depression (PROMIS SF4a T-score ≥ 50) | 1.68 (1.23-2.29) | 0.159 | 0.001 |
| Anxiety (PROMIS SF4a T-score ≥ 50) | 1.38 (1.02-1.87) | 0.155 | 0.038 |
| Sleep Disturbance (PROMIS SF4a T-score ≥ 50) | 1.96 (1.44-2.66) | 0.157 | <0.001 |
| Extraversion (TIPI ≥ 5.0) | 0.95 (0.70-1.30) | 0.158 | 0.763 |
| Agreeableness (TIPI ≥ 5.0) | 0.90 (0.65-1.23) | 0.161 | 0.502 |
| Conscientiousness (TIPI ≥ 5.0) | 0.88 (0.60-1.30) | 0.199 | 0.528 |
| Emotional Stability (TIPI ≥ 5.0) | 0.71 (0.53-0.97) | 0.154 | 0.029 |
| Open to New Experiences (TIPI ≥ 5.0) | 1.24 (0.91-1.67) | 0.154 | 0.168 |
| Traumatic events before 18 years of age | 1.81 (1.34-2.45) | 0.154 | <0.001 |
| Hospital stay before 18 years of age | 1.08 (0.56-2.09) | 0.336 | 0.818 |
| Ever smoked | 1.64 (1.22-2.20) | 0.151 | 0.001 |
| Currently drink alcohol | 0.85 (0.52-1.39) | 0.251 | 0.525 |
| Physical Activity (High) | 0.86 (0.59-1.25) | 0.188 | 0.426 |
| Health-related quality of life (EQ-5D-5L Index ≥ 0.800) | 0.29 (0.22-0.40) | 0.154 | <0.001 |
| BMI (≥30kg/m^2^) | 1.54 (1.06-2.24) | 0.192 | 0.025 |
| Blood pressure (≥140/90 mmHg) | 1.17 (0.85-1.61) | 0.163 | 0.341 |
| Total cholesterol (≥5 mmol/L) | 0.96 (0.70-1.33) | 0.163 | 0.813 |

BMI, Body mass index; BPM, beats per minute; EQ-5D-5L, EuroQoL-five dimensions-five levels; GS:SFHS, Generation Scotland: Scottish Family Health Study; PROMIS, Patient-Reported Outcomes Measurement Information System; SF4a, short form four answers; SIMD, Scottish Index of Multiple Deprivation; TIPI, Ten Item Personality Inventory

# **Supplementary Table S3** - Univariate analysis of resolved neuropathic pain in GS:SFHS (pooled analysis after multiple imputation m = 59)

| **Characteristic** | **Odds Ratio (95% Confidence Interval)** | **Standard Error** | **P-value** |
| --- | --- | --- | --- |
| Age (≥65 years) | 0.90 (0.58-1.38) | 0.219 | 0.622 |
| Gender (Male) | 1.19 (0.79-1.77) | 0.204 | 0.401 |
| Social Deprivation (SIMD)  1 (Most deprived)  2  3  4  5 (Least deprived) | -  1.79 (0.83-3.86)  2.97 (1.34-6.58)  2.17 (1.04-4.50)  3.24 (1.59-6.57) | -  0.391  0.404  0.371  0.360 | -  0.137  0.007  0.038  0.001 |
| Depression (PROMIS T-score ≥ 50) | 0.50 (0.34-0.75) | 0.202 | <0.001 |
| Anxiety (PROMIS T-score ≥ 50) | 0.47 (0.31-0.70) | 0.205 | <0.001 |
| Sleep Disturbance (PROMIS T-score ≥ 50) | 0.40 (0.25-0.63) | 0.230 | <0.001 |
| Extraversion (TIPI ≥ 5.0) | 1.23 (0.82-1.85) | 0.208 | 0.321 |
| Agreeableness (TIPI ≥ 5.0) | 1.06 (0.71-1.60) | 0.207 | 0.764 |
| Conscientiousness (TIPI ≥ 5.0) | 3.28 (1.99-5.42) | 0.255 | <0.001 |
| Emotional Stability (TIPI ≥ 5.0) | 2.30 (1.55-3.42) | 0.202 | <0.001 |
| Open to New Experiences (TIPI ≥ 5.0) | 1.37 (0.93-2.03) | 0.198 | 0.109 |
| Pain-related worrying (PCS ≥ 30) | 0.41 (0.19-0.89) | 0.395 | 0.024 |
| Traumatic events before 18 years of age | 0.63 (0.42-0.93) | 0.202 | 0.021 |
| Hospital stay before 18 years of age | 1.25 (0.61-2.53) | 0.360 | 0.542 |
| Ever smoked | 0.64 (0.43-0.94) | 0.197 | 0.022 |
| Currently drink alcohol | 1.87 (1.05-3.34) | 0.294 | 0.033 |
| Physical Activity (High) | 0.67 (0.39-1.15) | 0.270 | 0.144 |
| Health-related quality of life (EQ-5D-5L Index ≥ 0.800) | 4.69 (2.74-8.04) | 0.274 | <0.001 |
| BMI (≥30 kg/m^2^) | 0.79 (0.52-1.21) | 0.213 | 0.280 |
| Blood pressure (≥140/90mmHg) | 1.09 (0.72-1.65) | 0.210 | 0.666 |
| Creatinine (≥100µmol/L) | 1.71 (0.72-4.05) | 0.439 | 0.223 |
| Total cholesterol (≥5mmol/L) | 0.79 (0.53-1.19) | 0.205 | 0.261 |
| High Density Lipoprotein (≥1mmol/L) | 1.11 (0.52-2.37) | 0.387 | 0.791 |

BMI, Body mass index; BPM, beats per minute; EQ-5D-5L, EuroQoL-five dimensions-five levels; GS:SFHS, Generation Scotland: Scottish Family Health Study; PROMIS, Patient-Reported Outcomes Measurement Information System; SF4a, short form four answers; SIMD, Scottish Index of Multiple Deprivation; TIPI, Ten Item Personality Inventory

# **Supplementary Table S4.** Internal validation performance metrics in each imputed dataset for the risk model for incident neuropathic pain

| **Dataset** | **Performance** | **Discrimination** | | **Calibration** | |
| --- | --- | --- | --- | --- | --- |
|  | **Nagelkerke R^2^** | **AUROC** | **AUPRC** | **Slope** | **Intercept** |
| **1** | 0.091 | 0.719 | 0.128 | 1.026 | 0.026 |
| **2** | 0.092 | 0.720 | 0.133 | 1.037 | 0.042 |
| **3** | 0.089 | 0.714 | 0.132 | 1.019 | -0.007 |
| **4** | 0.090 | 0.720 | 0.126 | 1.026 | 0.017 |
| **5** | 0.090 | 0.721 | 0.127 | 1.033 | 0.048 |
| **6** | 0.089 | 0.718 | 0.125 | 1.018 | -0.007 |
| **7** | 0.085 | 0.716 | 0.123 | 0.994 | -0.054 |
| **8** | 0.086 | 0.716 | 0.125 | 1.007 | -0.019 |
| **9** | 0.093 | 0.720 | 0.130 | 1.055 | 0.095 |
| **10** | 0.093 | 0.720 | 0.130 | 1.053 | 0.099 |
| **11** | 0.098 | 0.727 | 0.138 | 1.084 | 0.163 |
| **12** | 0.084 | 0.712 | 0.126 | 1.004 | -0.025 |
| **13** | 0.086 | 0.716 | 0.125 | 1.002 | -0.032 |
| **14** | 0.093 | 0.724 | 0.127 | 1.047 | 0.070 |
| **15** | 0.079 | 0.708 | 0.120 | 0.957 | -0.157 |
| **16** | 0.092 | 0.720 | 0.127 | 1.032 | 0.043 |
| **17** | 0.084 | 0.714 | 0.126 | 0.980 | -0.099 |
| **18** | 0.084 | 0.714 | 0.123 | 0.991 | -0.065 |
| **19** | 0.090 | 0.719 | 0.135 | 1.030 | 0.039 |
| **20** | 0.092 | 0.720 | 0.126 | 1.033 | 0.040 |
| **21** | 0.093 | 0.720 | 0.135 | 1.047 | 0.069 |
| **22** | 0.096 | 0.722 | 0.138 | 1.072 | 0.138 |
| **23** | 0.085 | 0.713 | 0.127 | 0.988 | -0.080 |
| **24** | 0.098 | 0.727 | 0.139 | 1.080 | 0.155 |
| **25** | 0.094 | 0.722 | 0.130 | 1.054 | 0.094 |
| **26** | 0.087 | 0.714 | 0.132 | 1.011 | -0.010 |
| **27** | 0.092 | 0.721 | 0.129 | 1.043 | 0.059 |
| **28** | 0.096 | 0.727 | 0.129 | 1.061 | 0.113 |
| **29** | 0.091 | 0.720 | 0.128 | 1.042 | 0.060 |
| **30** | 0.092 | 0.721 | 0.127 | 1.036 | 0.045 |
| **31** | 0.093 | 0.721 | 0.130 | 1.056 | 0.099 |
| **32** | 0.095 | 0.726 | 0.129 | 1.060 | 0.109 |
| **33** | 0.093 | 0.722 | 0.130 | 1.053 | 0.092 |
| **34** | 0.087 | 0.714 | 0.129 | 1.025 | 0.034 |
| **35** | 0.092 | 0.720 | 0.127 | 1.040 | 0.057 |
| **36** | 0.093 | 0.722 | 0.129 | 1.048 | 0.081 |
| **37** | 0.090 | 0.719 | 0.125 | 1.027 | 0.020 |
| **38** | 0.091 | 0.721 | 0.126 | 1.033 | 0.039 |
| **39** | 0.090 | 0.717 | 0.132 | 1.021 | 0.004 |
| **40** | 0.091 | 0.721 | 0.127 | 1.034 | 0.041 |
| **41** | 0.088 | 0.716 | 0.123 | 1.005 | -0.041 |
| **42** | 0.089 | 0.718 | 0.132 | 1.023 | 0.022 |
| **43** | 0.085 | 0.715 | 0.125 | 1.004 | -0.033 |
| **44** | 0.089 | 0.717 | 0.127 | 1.025 | 0.018 |
| **45** | 0.095 | 0.725 | 0.133 | 1.056 | 0.087 |
| **46** | 0.090 | 0.720 | 0.133 | 1.028 | 0.025 |
| **47** | 0.095 | 0.724 | 0.130 | 1.056 | 0.105 |
| **48** | 0.096 | 0.723 | 0.138 | 1.065 | 0.117 |
| **49** | 0.089 | 0.716 | 0.127 | 1.022 | 0.011 |
| **50** | 0.082 | 0.710 | 0.123 | 0.978 | -0.096 |
| **51** | 0.096 | 0.726 | 0.131 | 1.070 | 0.133 |
| **52** | 0.090 | 0.718 | 0.127 | 1.028 | 0.030 |
| **53** | 0.082 | 0.710 | 0.124 | 0.987 | -0.073 |
| **54** | 0.090 | 0.719 | 0.136 | 1.035 | 0.052 |
| **55** | 0.083 | 0.712 | 0.127 | 0.980 | -0.105 |
| **56** | 0.089 | 0.716 | 0.127 | 1.025 | 0.019 |
| **57** | 0.097 | 0.725 | 0.137 | 1.068 | 0.129 |
| **58** | 0.095 | 0.724 | 0.131 | 1.060 | 0.113 |
| **59** | 0.093 | 0.721 | 0.131 | 1.056 | 0.099 |
| **60** | 0.091 | 0.721 | 0.127 | 1.034 | 0.039 |

AUPRC, area under the precision-recall curve; AUROC, area under the receiver operating characteristic curve

# **Supplementary Table S5.** External validation performance metrics in each imputed dataset for the risk model for incident neuropathic pain

| **Dataset** | **Performance** | **Discrimination** | | **Calibration** | | **Clinical Utility** |
| --- | --- | --- | --- | --- | --- | --- |
|  | **Nagelkerke R^2^** | **AUROC** | **AUPRC** | **Slope** | **Intercept** | **Net Benefit*** |
| **1** | 0.036 | 0.629 | 0.150 | 0.600 | -0.563 | 0.022 |
| **2** | 0.042 | 0.646 | 0.153 | 0.653 | -0.441 | 0.023 |
| **3** | 0.051 | 0.659 | 0.163 | 0.718 | -0.278 | 0.024 |
| **4** | 0.034 | 0.626 | 0.146 | 0.576 | -0.636 | 0.022 |
| **5** | 0.039 | 0.638 | 0.149 | 0.636 | -0.484 | 0.022 |
| **6** | 0.044 | 0.645 | 0.156 | 0.670 | -0.395 | 0.023 |
| **7** | 0.047 | 0.647 | 0.166 | 0.689 | -0.351 | 0.022 |
| **8** | 0.043 | 0.643 | 0.155 | 0.666 | -0.411 | 0.022 |
| **9** | 0.040 | 0.638 | 0.157 | 0.640 | -0.460 | 0.022 |
| **10** | 0.045 | 0.649 | 0.155 | 0.686 | -0.353 | 0.023 |
| **11** | 0.034 | 0.630 | 0.153 | 0.582 | -0.612 | 0.019 |
| **12** | 0.034 | 0.633 | 0.148 | 0.587 | -0.595 | 0.020 |
| **13** | 0.031 | 0.625 | 0.145 | 0.554 | -0.689 | 0.020 |
| **14** | 0.036 | 0.632 | 0.148 | 0.604 | -0.558 | 0.021 |
| **15** | 0.039 | 0.638 | 0.151 | 0.624 | -0.506 | 0.022 |
| **16** | 0.032 | 0.623 | 0.146 | 0.561 | -0.660 | 0.021 |
| **17** | 0.048 | 0.650 | 0.163 | 0.704 | -0.309 | 0.024 |
| **18** | 0.030 | 0.621 | 0.149 | 0.544 | -0.701 | 0.018 |
| **19** | 0.043 | 0.642 | 0.158 | 0.658 | -0.422 | 0.022 |
| **20** | 0.046 | 0.654 | 0.159 | 0.694 | -0.341 | 0.023 |
| **21** | 0.034 | 0.625 | 0.153 | 0.591 | -0.595 | 0.021 |
| **22** | 0.036 | 0.630 | 0.147 | 0.602 | -0.562 | 0.023 |
| **23** | 0.039 | 0.637 | 0.154 | 0.634 | -0.478 | 0.022 |
| **24** | 0.041 | 0.638 | 0.164 | 0.651 | -0.441 | 0.021 |
| **25** | 0.033 | 0.627 | 0.151 | 0.577 | -0.615 | 0.020 |
| **26** | 0.032 | 0.624 | 0.147 | 0.565 | -0.649 | 0.020 |
| **27** | 0.035 | 0.628 | 0.149 | 0.592 | -0.593 | 0.021 |
| **28** | 0.049 | 0.656 | 0.159 | 0.714 | -0.290 | 0.024 |
| **29** | 0.039 | 0.638 | 0.153 | 0.624 | -0.511 | 0.021 |
| **30** | 0.039 | 0.639 | 0.152 | 0.631 | -0.491 | 0.021 |
| **31** | 0.037 | 0.633 | 0.147 | 0.615 | -0.532 | 0.022 |
| **32** | 0.037 | 0.632 | 0.150 | 0.608 | -0.558 | 0.022 |
| **33** | 0.033 | 0.629 | 0.146 | 0.581 | -0.609 | 0.020 |
| **34** | 0.041 | 0.641 | 0.150 | 0.647 | -0.443 | 0.024 |
| **35** | 0.033 | 0.630 | 0.146 | 0.585 | -0.604 | 0.019 |
| **36** | 0.039 | 0.635 | 0.152 | 0.623 | -0.505 | 0.022 |
| **37** | 0.044 | 0.644 | 0.157 | 0.664 | -0.407 | 0.024 |
| **38** | 0.048 | 0.650 | 0.164 | 0.702 | -0.321 | 0.023 |
| **39** | 0.045 | 0.649 | 0.156 | 0.682 | -0.367 | 0.023 |
| **40** | 0.042 | 0.643 | 0.151 | 0.657 | -0.430 | 0.022 |
| **41** | 0.036 | 0.636 | 0.147 | 0.600 | -0.561 | 0.019 |
| **42** | 0.037 | 0.635 | 0.149 | 0.609 | -0.547 | 0.021 |
| **43** | 0.036 | 0.629 | 0.154 | 0.612 | -0.549 | 0.020 |
| **44** | 0.042 | 0.641 | 0.157 | 0.659 | -0.426 | 0.022 |
| **45** | 0.042 | 0.643 | 0.163 | 0.660 | -0.425 | 0.020 |
| **46** | 0.036 | 0.635 | 0.147 | 0.602 | -0.561 | 0.022 |
| **47** | 0.040 | 0.642 | 0.151 | 0.634 | -0.485 | 0.021 |
| **48** | 0.032 | 0.625 | 0.148 | 0.569 | -0.646 | 0.021 |
| **49** | 0.037 | 0.631 | 0.150 | 0.607 | -0.553 | 0.022 |
| **50** | 0.037 | 0.635 | 0.151 | 0.616 | -0.530 | 0.021 |
| **51** | 0.047 | 0.649 | 0.160 | 0.692 | -0.344 | 0.023 |
| **52** | 0.039 | 0.639 | 0.152 | 0.633 | -0.482 | 0.021 |
| **53** | 0.033 | 0.627 | 0.147 | 0.576 | -0.635 | 0.020 |
| **54** | 0.047 | 0.653 | 0.157 | 0.690 | -0.343 | 0.024 |
| **55** | 0.046 | 0.646 | 0.158 | 0.686 | -0.350 | 0.024 |
| **56** | 0.035 | 0.628 | 0.157 | 0.597 | -0.570 | 0.020 |
| **57** | 0.035 | 0.629 | 0.152 | 0.592 | -0.584 | 0.020 |
| **58** | 0.042 | 0.640 | 0.160 | 0.650 | -0.441 | 0.022 |
| **59** | 0.035 | 0.629 | 0.153 | 0.587 | -0.596 | 0.020 |
| **60** | 0.028 | 0.617 | 0.145 | 0.525 | -0.756 | 0.018 |
| **61** | 0.036 | 0.638 | 0.148 | 0.613 | -0.539 | 0.021 |
| **62** | 0.039 | 0.634 | 0.156 | 0.628 | -0.498 | 0.022 |
| **63** | 0.038 | 0.632 | 0.150 | 0.615 | -0.527 | 0.023 |
| **64** | 0.038 | 0.637 | 0.152 | 0.623 | -0.511 | 0.021 |
| **65** | 0.029 | 0.623 | 0.146 | 0.540 | -0.715 | 0.017 |

AUPRC, area under the precision-recall curve; AUROC, area under the receiver operating characteristic curve

***At incidence threshold: 10.7%**

# **Supplementary Table S6.** Internal validation performance metrics in each imputed dataset for the risk model for resolved neuropathic pain

| **Dataset** | **Performance** | **Discrimination** | | **Calibration** | |
| --- | --- | --- | --- | --- | --- |
|  | **Nagelkerke R^2^** | **AUROC** | **AUPRC** | **Slope** | **Intercept** |
| **1** | 0.271 | 0.746 | 0.763 | 1.201 | 0.159 |
| **2** | 0.286 | 0.746 | 0.761 | 1.180 | 0.215 |
| **3** | 0.317 | 0.759 | 0.779 | 1.321 | 0.260 |
| **4** | 0.296 | 0.748 | 0.764 | 1.260 | 0.215 |
| **5** | 0.296 | 0.761 | 0.771 | 1.227 | 0.166 |
| **6** | 0.320 | 0.775 | 0.785 | 1.329 | 0.192 |
| **7** | 0.270 | 0.743 | 0.753 | 1.142 | 0.184 |
| **8** | 0.264 | 0.732 | 0.749 | 1.173 | 0.165 |
| **9** | 0.310 | 0.766 | 0.779 | 1.296 | 0.231 |
| **10** | 0.280 | 0.749 | 0.774 | 1.149 | 0.134 |
| **11** | 0.262 | 0.739 | 0.762 | 1.142 | 0.185 |
| **12** | 0.275 | 0.746 | 0.759 | 1.141 | 0.226 |
| **13** | 0.277 | 0.745 | 0.771 | 1.226 | 0.184 |
| **14** | 0.319 | 0.772 | 0.776 | 1.319 | 0.241 |
| **15** | 0.266 | 0.730 | 0.748 | 1.177 | 0.150 |
| **16** | 0.286 | 0.747 | 0.760 | 1.204 | 0.196 |
| **17** | 0.356 | 0.792 | 0.794 | 1.435 | 0.233 |
| **18** | 0.286 | 0.762 | 0.777 | 1.202 | 0.172 |
| **19** | 0.267 | 0.738 | 0.753 | 1.168 | 0.153 |
| **20** | 0.303 | 0.761 | 0.776 | 1.230 | 0.191 |
| **21** | 0.289 | 0.753 | 0.768 | 1.259 | 0.168 |
| **22** | 0.300 | 0.771 | 0.779 | 1.265 | 0.159 |
| **23** | 0.313 | 0.763 | 0.792 | 1.285 | 0.206 |
| **24** | 0.287 | 0.758 | 0.773 | 1.217 | 0.212 |
| **25** | 0.285 | 0.754 | 0.773 | 1.217 | 0.164 |
| **26** | 0.369 | 0.800 | 0.799 | 1.487 | 0.258 |
| **27** | 0.301 | 0.760 | 0.774 | 1.262 | 0.179 |
| **28** | 0.300 | 0.755 | 0.763 | 1.288 | 0.188 |
| **29** | 0.313 | 0.765 | 0.789 | 1.277 | 0.228 |
| **30** | 0.332 | 0.777 | 0.794 | 1.401 | 0.197 |
| **31** | 0.278 | 0.753 | 0.768 | 1.177 | 0.196 |
| **32** | 0.315 | 0.770 | 0.788 | 1.305 | 0.209 |
| **33** | 0.265 | 0.739 | 0.749 | 1.142 | 0.155 |
| **34** | 0.293 | 0.762 | 0.775 | 1.207 | 0.102 |
| **35** | 0.328 | 0.770 | 0.780 | 1.357 | 0.219 |
| **36** | 0.271 | 0.745 | 0.765 | 1.141 | 0.146 |
| **37** | 0.255 | 0.736 | 0.760 | 1.134 | 0.194 |
| **38** | 0.363 | 0.795 | 0.809 | 1.444 | 0.241 |
| **39** | 0.292 | 0.760 | 0.765 | 1.283 | 0.157 |
| **40** | 0.277 | 0.745 | 0.770 | 1.148 | 0.131 |
| **41** | 0.317 | 0.769 | 0.781 | 1.262 | 0.225 |
| **42** | 0.281 | 0.750 | 0.763 | 1.184 | 0.150 |
| **43** | 0.272 | 0.747 | 0.772 | 1.169 | 0.105 |
| **44** | 0.239 | 0.725 | 0.749 | 1.019 | 0.114 |
| **45** | 0.256 | 0.740 | 0.727 | 1.094 | 0.113 |
| **46** | 0.318 | 0.769 | 0.783 | 1.345 | 0.143 |
| **47** | 0.305 | 0.766 | 0.781 | 1.262 | 0.234 |
| **48** | 0.288 | 0.754 | 0.770 | 1.252 | 0.172 |
| **49** | 0.289 | 0.756 | 0.778 | 1.170 | 0.168 |
| **50** | 0.295 | 0.756 | 0.765 | 1.216 | 0.161 |
| **51** | 0.303 | 0.758 | 0.784 | 1.269 | 0.157 |
| **52** | 0.272 | 0.744 | 0.758 | 1.202 | 0.161 |
| **53** | 0.302 | 0.765 | 0.787 | 1.257 | 0.217 |
| **54** | 0.263 | 0.742 | 0.756 | 1.084 | 0.081 |
| **55** | 0.280 | 0.752 | 0.761 | 1.193 | 0.186 |
| **56** | 0.301 | 0.766 | 0.775 | 1.277 | 0.223 |
| **57** | 0.307 | 0.757 | 0.778 | 1.294 | 0.205 |
| **58** | 0.318 | 0.767 | 0.785 | 1.310 | 0.267 |
| **59** | 0.310 | 0.768 | 0.798 | 1.297 | 0.255 |
| **60** | 0.285 | 0.752 | 0.766 | 1.236 | 0.189 |
| **61** | 0.314 | 0.767 | 0.785 | 1.321 | 0.182 |
| **62** | 0.282 | 0.754 | 0.770 | 1.164 | 0.141 |
| **63** | 0.300 | 0.755 | 0.763 | 1.220 | 0.179 |
| **64** | 0.291 | 0.760 | 0.774 | 1.185 | 0.133 |
| **65** | 0.277 | 0.750 | 0.760 | 1.195 | 0.167 |
| **66** | 0.315 | 0.764 | 0.784 | 1.289 | 0.214 |

AUPRC, area under the precision-recall curve; AUROC, area under the receiver operating characteristic curve

# **Supplementary Table S7.** External validation performance metrics in each imputed dataset for the risk model for resolved neuropathic pain

| **Dataset** | **Performance** | **Discrimination** | | **Calibration** | | **Clinical Utility** |
| --- | --- | --- | --- | --- | --- | --- |
|  | **Nagelkerke R^2^** | **AUROC** | **AUPRC** | **Slope** | **Intercept** | **Net Benefit*** |
| **1** | 0.148 | 0.702 | 0.480 | 0.973 | -0.436 | 0.065 |
| **2** | 0.152 | 0.698 | 0.522 | 1.034 | -0.383 | 0.061 |
| **3** | 0.162 | 0.710 | 0.508 | 1.096 | -0.351 | 0.063 |
| **4** | 0.149 | 0.696 | 0.502 | 0.998 | -0.419 | 0.058 |
| **5** | 0.164 | 0.708 | 0.513 | 1.064 | -0.370 | 0.075 |
| **6** | 0.140 | 0.697 | 0.472 | 0.974 | -0.440 | 0.059 |
| **7** | 0.173 | 0.724 | 0.505 | 1.062 | -0.417 | 0.083 |
| **8** | 0.160 | 0.718 | 0.495 | 1.037 | -0.450 | 0.079 |
| **9** | 0.128 | 0.689 | 0.457 | 0.894 | -0.476 | 0.054 |
| **10** | 0.160 | 0.711 | 0.499 | 1.051 | -0.395 | 0.082 |
| **11** | 0.139 | 0.696 | 0.479 | 0.911 | -0.497 | 0.061 |
| **12** | 0.175 | 0.717 | 0.526 | 1.096 | -0.338 | 0.079 |
| **13** | 0.133 | 0.686 | 0.510 | 0.942 | -0.455 | 0.065 |
| **14** | 0.146 | 0.698 | 0.502 | 1.007 | -0.424 | 0.059 |
| **15** | 0.146 | 0.698 | 0.502 | 1.004 | -0.422 | 0.075 |
| **16** | 0.171 | 0.717 | 0.516 | 1.098 | -0.365 | 0.076 |
| **17** | 0.176 | 0.725 | 0.517 | 1.132 | -0.317 | 0.078 |
| **18** | 0.143 | 0.694 | 0.486 | 0.971 | -0.454 | 0.061 |
| **19** | 0.137 | 0.697 | 0.484 | 0.980 | -0.403 | 0.075 |
| **20** | 0.174 | 0.722 | 0.516 | 1.104 | -0.367 | 0.076 |
| **21** | 0.140 | 0.696 | 0.494 | 0.950 | -0.478 | 0.061 |
| **22** | 0.166 | 0.717 | 0.516 | 1.062 | -0.387 | 0.073 |
| **23** | 0.135 | 0.689 | 0.479 | 0.949 | -0.480 | 0.060 |
| **24** | 0.133 | 0.686 | 0.480 | 0.947 | -0.447 | 0.066 |
| **25** | 0.152 | 0.699 | 0.505 | 0.997 | -0.418 | 0.065 |
| **26** | 0.160 | 0.709 | 0.497 | 1.027 | -0.408 | 0.068 |
| **27** | 0.154 | 0.707 | 0.500 | 1.028 | -0.427 | 0.061 |
| **28** | 0.154 | 0.698 | 0.524 | 1.038 | -0.417 | 0.067 |
| **29** | 0.132 | 0.681 | 0.497 | 0.931 | -0.480 | 0.051 |
| **30** | 0.148 | 0.703 | 0.512 | 0.993 | -0.454 | 0.064 |
| **31** | 0.134 | 0.693 | 0.465 | 0.926 | -0.483 | 0.064 |
| **32** | 0.134 | 0.697 | 0.474 | 0.971 | -0.449 | 0.065 |
| **33** | 0.147 | 0.708 | 0.470 | 0.976 | -0.445 | 0.079 |
| **34** | 0.172 | 0.716 | 0.523 | 1.077 | -0.394 | 0.074 |
| **35** | 0.121 | 0.678 | 0.470 | 0.886 | -0.521 | 0.059 |
| **36** | 0.140 | 0.694 | 0.490 | 0.929 | -0.514 | 0.067 |
| **37** | 0.163 | 0.704 | 0.517 | 1.053 | -0.393 | 0.069 |
| **38** | 0.152 | 0.702 | 0.485 | 1.000 | -0.426 | 0.069 |
| **39** | 0.136 | 0.702 | 0.454 | 0.950 | -0.455 | 0.067 |
| **40** | 0.162 | 0.711 | 0.502 | 1.037 | -0.413 | 0.065 |
| **41** | 0.142 | 0.695 | 0.498 | 0.990 | -0.382 | 0.062 |
| **42** | 0.150 | 0.700 | 0.505 | 1.021 | -0.385 | 0.060 |
| **43** | 0.145 | 0.697 | 0.484 | 0.982 | -0.448 | 0.067 |
| **44** | 0.152 | 0.704 | 0.510 | 1.018 | -0.394 | 0.069 |
| **45** | 0.171 | 0.715 | 0.516 | 1.095 | -0.331 | 0.067 |
| **46** | 0.139 | 0.692 | 0.479 | 0.965 | -0.445 | 0.057 |
| **47** | 0.152 | 0.704 | 0.493 | 1.018 | -0.423 | 0.072 |
| **48** | 0.130 | 0.691 | 0.461 | 0.921 | -0.472 | 0.063 |
| **49** | 0.155 | 0.708 | 0.494 | 1.035 | -0.420 | 0.066 |
| **50** | 0.148 | 0.706 | 0.497 | 1.002 | -0.423 | 0.065 |
| **51** | 0.127 | 0.684 | 0.484 | 0.928 | -0.458 | 0.056 |
| **52** | 0.149 | 0.695 | 0.526 | 0.983 | -0.451 | 0.061 |
| **53** | 0.137 | 0.692 | 0.487 | 0.950 | -0.465 | 0.061 |
| **54** | 0.140 | 0.692 | 0.504 | 0.970 | -0.435 | 0.056 |
| **55** | 0.162 | 0.715 | 0.507 | 1.092 | -0.374 | 0.067 |
| **56** | 0.163 | 0.710 | 0.518 | 1.086 | -0.395 | 0.068 |

AUPRC, area under the precision-recall curve; AUROC, area under the receiver operating characteristic curve

***At incidence threshold: 23.7%**


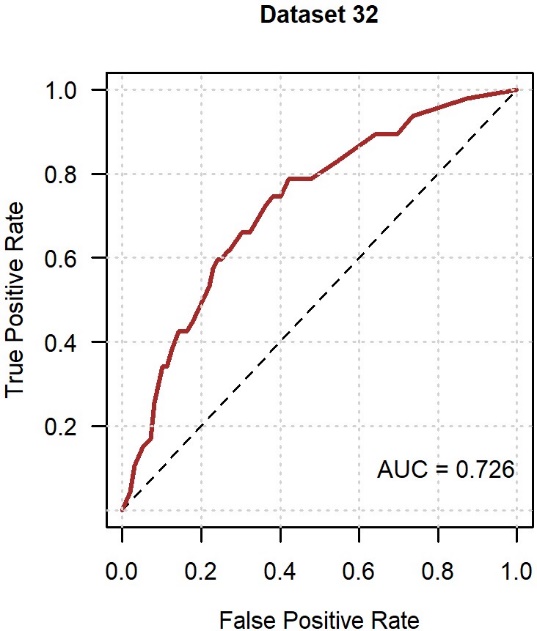


# **Supplementary Figure S1.** ROC curve for internal validation of the risk model for incident neuropathic pain (imputed dataset 32)


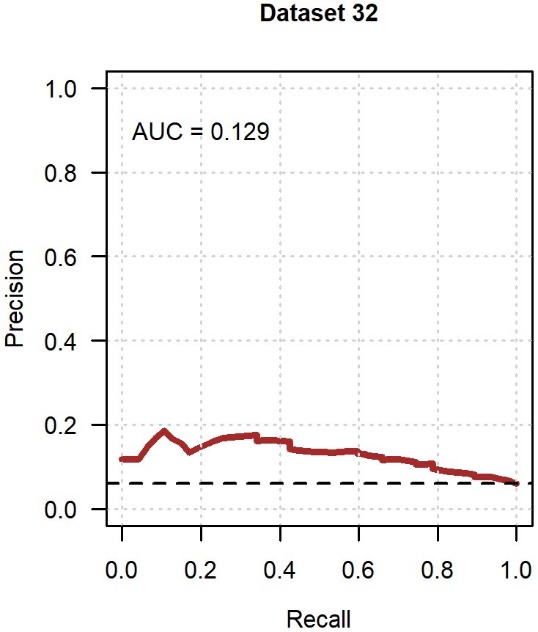


# **Supplementary Figure S2.** Precision-recall curve for internal validation of the risk model for incident neuropathic pain (imputed dataset 32)


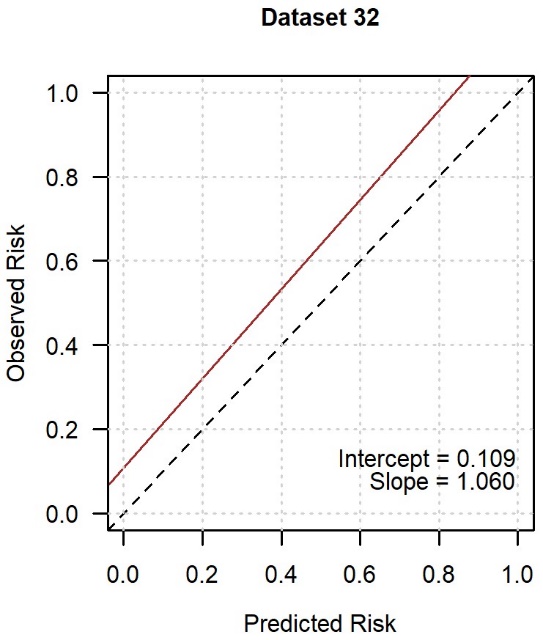


# **Supplementary Figure S3.** Calibration curve for internal validation of the risk model for incident neuropathic pain (imputed dataset 32)


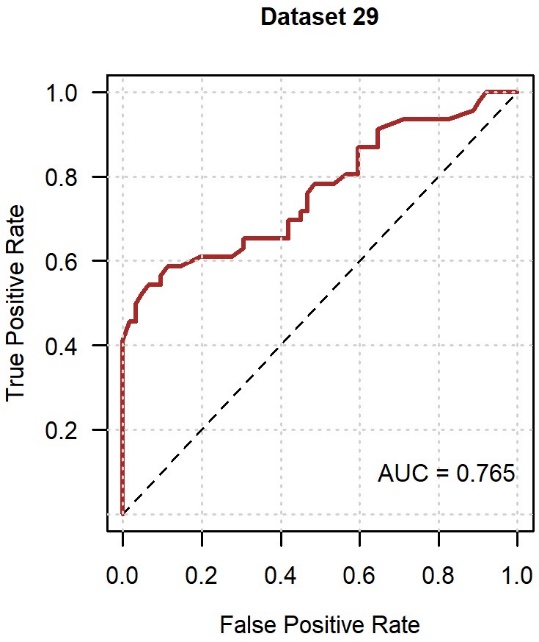


# **Supplementary Figure S4.** ROC curve for internal validation of the risk model for resolved neuropathic pain (imputed dataset 29)


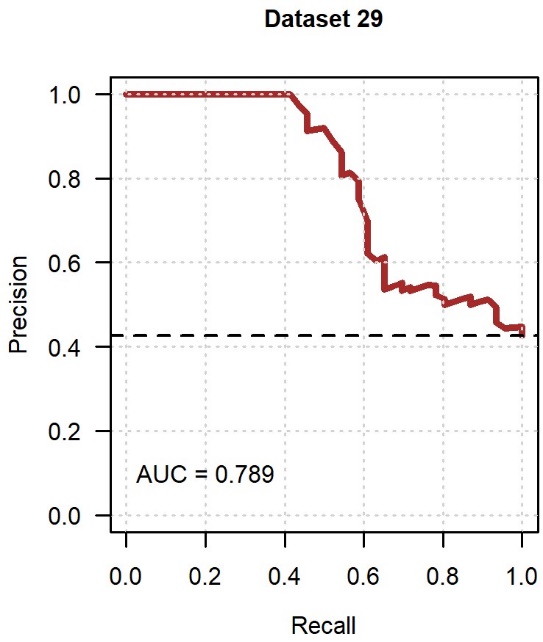


# **Supplementary Figure S5.** Precision-recall curve for internal validation of the risk model for resolved neuropathic pain (imputed dataset 29)


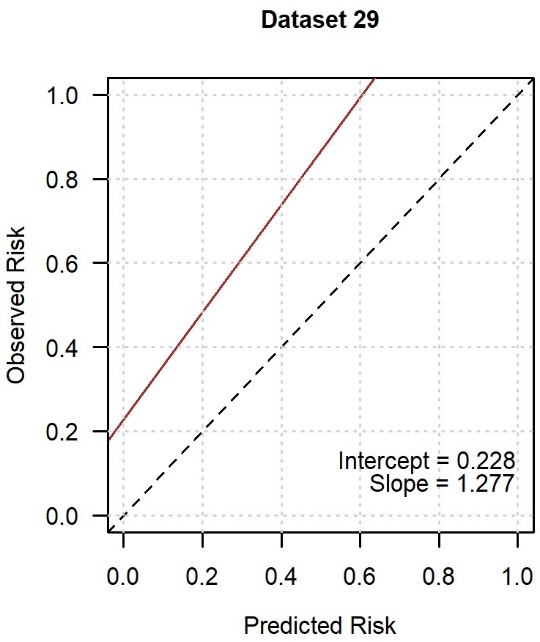


# **Supplementary Figure S6.** Calibration curve for internal validation of the risk model for resolved neuropathic pain (imputed dataset 29)
